# Analysis of endothelial-to-haematopoietic transition at the single cell level identifies cell cycle regulation as a driver of differentiation

**DOI:** 10.1186/s13059-020-02058-4

**Published:** 2020-07-01

**Authors:** Giovanni Canu, Emmanouil Athanasiadis, Rodrigo A. Grandy, Jose Garcia-Bernardo, Paulina M. Strzelecka, Ludovic Vallier, Daniel Ortmann, Ana Cvejic

**Affiliations:** 1 Wellcome Trust–Medical Research Council Cambridge Stem Cell Institute, Cambridge, UK; 2 Department of Surgery, University of Cambridge, Cambridge, UK; 3 Department of Haematology, University of Cambridge, Cambridge, UK; 4 Wellcome Trust Sanger Institute, Wellcome Trust Genome Campus, Cambridge, UK; 5 GSK, Medicines Research Centre, Gunnels Wood Road, Stevenage, Hertfordshire, SG1 2NY UK

## Abstract

**Background:**

Haematopoietic stem cells (HSCs) first arise during development in the aorta-gonad-mesonephros (AGM) region of the embryo from a population of haemogenic endothelial cells which undergo endothelial-to-haematopoietic transition (EHT). Despite the progress achieved in recent years, the molecular mechanisms driving EHT are still poorly understood, especially in human where the AGM region is not easily accessible.

**Results:**

In this study, we take advantage of a human pluripotent stem cell (hPSC) differentiation system and single-cell transcriptomics to recapitulate EHT in vitro and uncover mechanisms by which the haemogenic endothelium generates early haematopoietic cells. We show that most of the endothelial cells reside in a quiescent state and progress to the haematopoietic fate within a defined time window, within which they need to re-enter into the cell cycle. If cell cycle is blocked, haemogenic endothelial cells lose their EHT potential and adopt a non-haemogenic identity. Furthermore, we demonstrate that CDK4/6 and CDK1 play a key role not only in the transition but also in allowing haematopoietic progenitors to establish their full differentiation potential.

**Conclusion:**

We propose a direct link between the molecular machineries that control cell cycle progression and EHT.

## Background

The first self-renewing haematopoietic stem cells (HSCs) are generated from the haemogenic endothelium, a specialised population of endothelial cells, located in the aorta-gonad-mesonephros (AGM) region [1–3]. This process is known as endothelial-to-haematopoietic transition (EHT) and is characterised by the appearance of intra-aortic haematopoietic clusters (IAHCs). IAHCs are physically associated with the haemogenic endothelium which is lining the ventral wall of the dorsal aorta in human [4, 5]. One of the first events that precedes EHT is the expression of RUNX1 in a subset of endothelial cells. Thus, RUNX1 expression marks the haemogenic endothelium where IAHCs will subsequently emerge [6]. It has been shown that RUNX1 activates the haematopoietic programme and at the same time mediates the upregulation of transcription factors (e.g. GFI1 and GFI1B) which in turn repress endothelial genes [7]. This dual role of RUNX1 possibly depends on its crosstalk with other key regulators of haematopoiesis such as TAL1 and GATA2 [8, 9]. In addition to the AGM, other secondary sites have been reported to produce HSCs from haemogenic endothelial cells through EHT later on during development, such as placenta, vitelline/umbilical arteries, and embryonic head [5, 10–14]. These first HSCs migrate to the foetal liver where their number dramatically increases, both as a consequence of proliferation and due to the contribution of secondary haematopoietic sites [5, 14]. Despite its importance, the mechanisms controlling EHT remain to be fully uncovered, especially in human where these developmental stages are difficult to access for obvious ethical reasons. To bypass these limitations, several groups have developed in vitro methods that recapitulate production of haematopoietic cells through the generation of an intermediate endothelial state [15–21].

Here, we took advantage of human pluripotent stem cells (hPSCs) to model haematopoietic development in vitro and used single-cell transcriptomics to dissect this process. We show that distinct populations are generated during EHT, including a population of haematopoietic progenitor cells that have multilineage differentiation potential. Furthermore, we demonstrated a tight link between cell cycle progression and EHT. Indeed, endothelial cells are quiescent and re-enter cell cycle to differentiate into haematopoietic progenitor cells. Inhibition of the cell cycle blocks EHT and causes endothelial cells to lose haemogenic potential. Finally, we demonstrated that cell cycle regulators such as CDK4/6 and CDK1 are not only essential for EHT but also control the capacity of nascent haematopoietic progenitors to differentiate. Together, our results uncover new mechanisms controlling the production of definitive haematopoietic cells which will be essential not only to understand blood cell development but also to improve protocols for generating these cells in vitro.

## Results

### hPSC differentiation provides an in vitro model of endothelial-to-haematopoietic transition

**Fig. 1:**
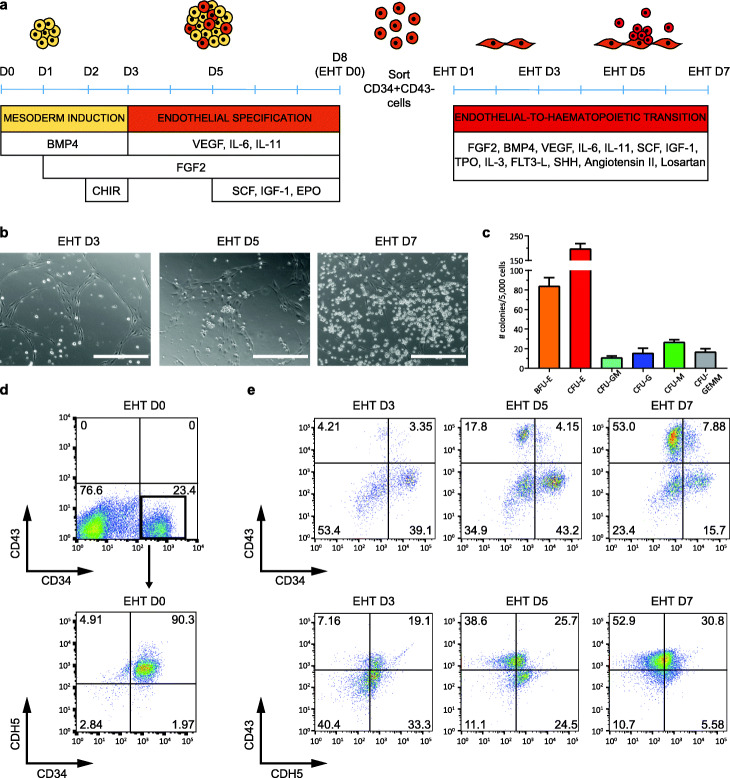
Culture system for modelling EHT in vitro. **a** Schematic of the differentiation protocol used to model EHT with hPSCs. D, day. **b** Representative images of cells progressing through EHT. Scale bar is 400 μm. **c** Quantification of colonies generated in a CFU assay using cells collected at EHT D5. Data represent means ± SEM of 3 independent experiments. BFU-E, burst forming unit-erythroid; CFU-E, colony forming unit-erythroid; CFU-GM, colony forming unit-granulocyte-macrophage; CFU-G, colony forming unit-granulocyte; CFU-M, colony forming unit-macrophage; CFU-GEMM, colony forming unit-granulocyte-erythrocyte-macrophage-megakaryocyte. **d**, **e** Flow cytometry analysis of **d** cells at D8/EHT D0 and **e** during EHT culture at multiple time points. Results are representative of at least 3 independent experiments

In order to gain insight into mechanisms driving human definitive haematopoiesis, we utilised a system for the differentiation of hPSCs (Fig. 1a) [22, 23]. This in vitro system recapitulates a natural path of development that leads to the production of an intermediate population of endothelial cells with haemogenic potential. Between EHT day 3 (D3) and EHT day 5 (D5), these endothelial cells generate round clusters that progressively increase in size and release single haematopoietic cells in the culture medium (Fig. 1b). Importantly, these haematopoietic cells can further differentiate into myeloid cells, foetal γ-globin-producing erythroid cells (Fig. 1c, Additional file 1: Fig. S1a, b), and T lymphocytes [23–25]. Transcriptionally, the process is marked by the gradual downregulation of endothelial markers (e.g. *CDH5*, *VWF*) and concomitant upregulation of blood markers (e.g. *SPI1*, *KLF1*), with key haematopoietic stem/progenitor cell (HSPC) genes peaking at EHT D3 (e.g. *RUNX1*, *MYB*, and *GATA2*) (Additional file 1: Fig. S1c, Fig. 1d, e). Taken together, our data show that differentiation of hPSCs morphologically resembles the generation of IAHCs [1, 2, 5, 25] and that the production of haematopoietic progenitors in vitro culminates around 3–5 days of EHT culture (Additional file 1: Fig. S1c). For the aforementioned reasons, we selected this interval for our subsequent analyses.

### Single-cell analysis revealed four distinct populations during in vitro EHT

**Fig. 2:**
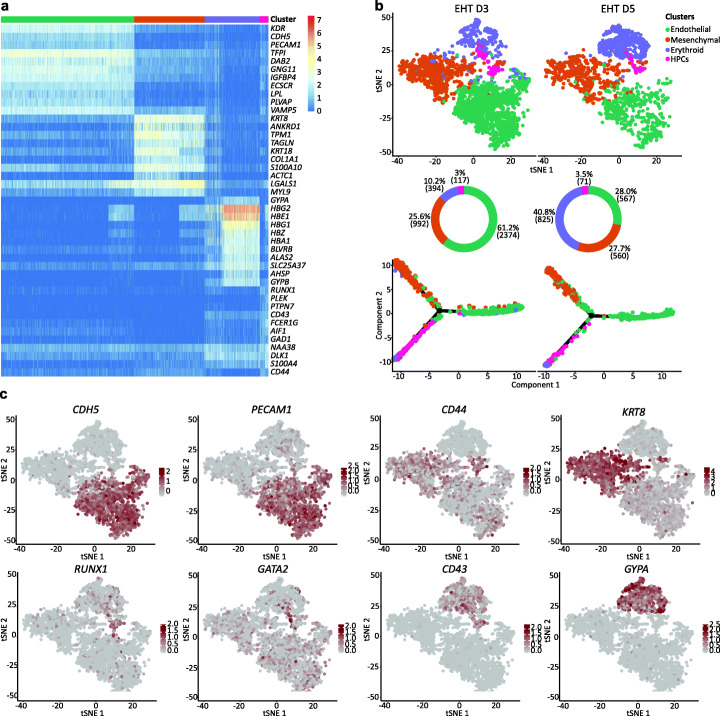
scRNA-seq reveals distinct cell types generated during hPSC differentiation. **a** Heatmap of the marker genes for each cluster. For this analysis, data from EHT D3 and EHT D5 were merged. **b** tSNE plots showing comparison of samples collected at EHT D3 and EHT D5. Four populations were identified based on transcriptional identity. Pie charts are showing the percentage and absolute number (in brackets) of cells in each cluster. Transcriptional data was used to order cells along differentiation trajectories, from endothelial cells (right) to the haematopoietic and mesenchymal branches (left). **c** tSNE plots of merged data from EHT D3 and EHT D5. Expression of relevant lineage markers is shown

EHT is a dynamic process with different cell types and states coexisting, thereby rendering bulk analyses difficult to interpret. To overcome this limitation and resolve the inherent heterogeneity of cell types generated during in vitro differentiation, we took advantage of single-cell RNA sequencing (scRNA-seq). In brief, cells were collected at EHT D3 and D5, processed using the Chromium 10X Genomics system, and analysed following the Seurat workflow [26]. Following quality control, 3877 cells from EHT D3 and 2152 from EHT D5 were used in downstream analyses. In both datasets, we identified four distinct populations (Fig. 2a, b) that were annotated based on marker genes and the presence of known lineage-specific signature genes (Fig. 2c, Additional file 1: Fig. S2a). Specifically, we identified a population of endothelial cells, positive for *CDH5* and *PECAM1*; mesenchymal cells, positive for *CDH2*, *KRT8*, and *TAGLN*; and two clusters of haematopoietic cells: haematopoietic progenitor cells (HPCs) and erythroid cells. Cells in the HPC cluster displayed expression of stem cell genes such as *RUNX1* and *GATA2*, as well as low expression of early erythroid and myeloid genes, e.g. *GATA1* and *CD33*. Cells in the erythroid cluster expressed erythroid lineage genes, e.g. *KLF1*, *ALAS2*, and *GYPA* (Fig. 2c, Additional file 1: Fig. S2a) and had immature cell morphology as shown by cytospin staining (Additional file 1: Fig. S2b) [27]. This cluster was therefore annotated as early erythroid progenitors.

To reconstruct the differentiation trajectory of haematopoietic cells during EHT, we compared cells from EHT D3 and EHT D5 (Fig. 2b). Our analyses revealed a marked reduction of endothelial cells and an increase of erythroid progenitors. Conversely, HPCs and mesenchymal cells only showed a minor increase. Pseudotime differentiation trajectories suggested that the haematopoietic and mesenchymal compartments both derived from a common endothelial population. Along this differentiation trajectory, endothelial cells gradually downregulated endothelial specific genes and concomitantly upregulated either mesenchymal or haematopoietic markers (Additional file 1: Fig. S3a). To validate the pseudotime differentiation trajectory, we sequenced further 1764 CD34+/CD43− cells sorted at EHT D0 (Additional file 1: Fig. S3b). Our analysis demonstrated that most cells at EHT D0 were endothelial, as shown by the positive expression of multiple endothelial marker genes (e.g. *CDH5*, *PECAM1*, *KDR*, *THY1*). Importantly, we did not observe expression of haematopoietic lineage associated genes, demonstrating that haematopoietic cells are subsequently generated. Accordingly, cells collected at EHT D0 were not able to generate blood colonies in a CFU assay. In addition, none of the identified clusters had a clear mesenchymal transcriptional signature. However, a small population co-expressed endothelial (e.g. *KDR*, *THY1*) and mesenchymal markers (e.g. *COL1A1*, *CDH2*, *CDH11*, *KRT8*) at low level, suggesting that a fraction of cells displayed a mixed endothelial/mesenchymal identity at EHT D0 and represented an early stage of mesenchymal commitment.

### Identification and functional validation of multipotent HPCs

Based on our scRNA-seq data, we devised a sorting strategy to isolate each of the populations identified above. We predicted that combinations of three cell surface markers (*CDH5*, *CD43*, and *CD44*) (Fig. 2c) were sufficient to allow sorting of the four populations. *CDH5* was mainly expressed in the endothelial cluster and to a lower extent in HPCs. The pan-haematopoietic *CD43* was expressed in HPCs and erythroid progenitors (Fig. 2c, Additional file 1: Fig. S2a). Both these genes are known to mark emerging HSPCs in the human dorsal aorta during development [28]. Finally, *CD44* appeared to be prevalently expressed in mesenchymal cells and HPCs (Fig. 2c). CD44 was previously shown to be expressed on IAHCs emerging during EHT in both human and mouse [29–31].

**Fig. 3:**
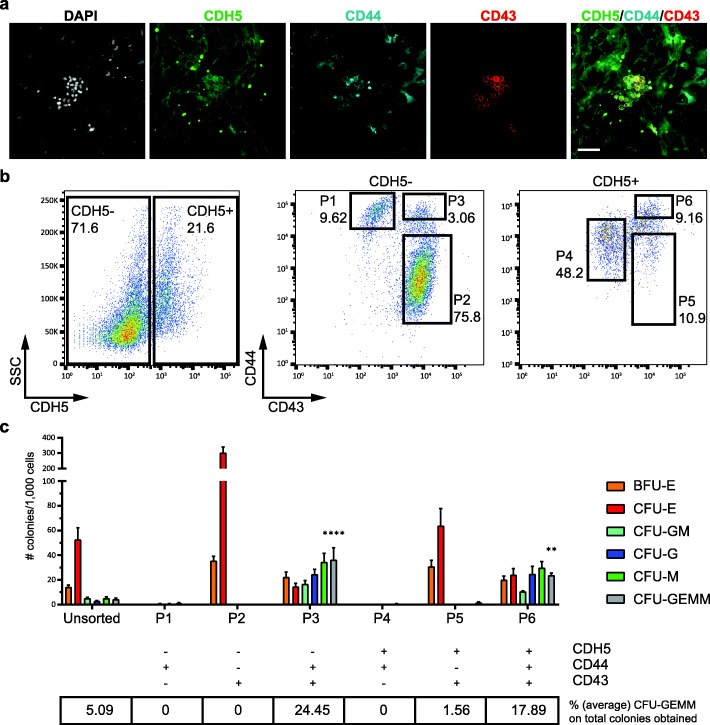
Sorting and functional validation of distinct populations. **a** Immunofluorescence staining showing expression of CDH5, CD44, and CD43 on haematopoietic clusters of cells at EHT D5. Scale bar is 50 μm. **b** Flow cytometry analysis showing the sorting strategy for six populations (P1–P6). Results are representative of at least 3 independent experiments, using 2 distinct hPSC lines. **c** CFU assay showing the differentiation potential of cells isolated from P1 to P6. The ability to produce multilineage CFU-GEMM colonies is compared to the unsorted population. Data are mean ± SEM of 4 independent experiments, with statistical significance compared to control as *P < 0.5, **P < 0.01, ***P < 0.001, and ****P < 0.0001 by one-way ANOVA. Degrees of freedom DFn = 6, DFd = 21, value *F* = 15.21

Immunostaining analyses confirmed that CDH5, CD43, and CD44 were co-expressed on the clusters of cells produced at EHT D5 (Fig. 3a). In addition, these cells expressed the master regulator of definitive haematopoiesis RUNX1 and were therefore likely to correspond to the HPC cluster identified in our single-cell analysis (Additional file 1: Fig. S4a). A lower expression of RUNX1 was also visible in some of the surrounding endothelial cells, possibly marking haemogenic endothelial cells in transition to the haematopoietic fate, consistent with what previously described in vivo [6].

Next, using combinations of the three cell surface markers, we isolated six distinct populations (Fig. 3b) which were assessed for their capacity to generate blood cells in a CFU assay (Fig. 3c). Cells in P1 (CDH5−/CD44+/CD43−) and P4 (CDH5+/CD44+/CD43−), predicted to label mesenchymal and endothelial populations, respectively, did not show haematopoietic potential in CFU assay. To further confirm their mesenchymal and endothelial identity, cells from P1 and P4 gates were re-plated and further cultured (Additional file 1: Fig. S4b-g). After 10 days in culture, more than half of the cells from P4 were CD31+/CD90+, consistent with an endothelial identity. Importantly, these cells were able to produce new mesenchymal (CDH5−/CD44high) and haematopoietic (CD43+) cells, confirming their differentiation potential. In contrast, cells sorted and re-plated from P1 gate displayed morphology and markers (CD44+/Vimentin+) consistent with a mesenchymal identity. These mesenchymal cells showed no ability to differentiate into endothelial cells (i.e. all cells were CDH5−). Furthermore, cell from the P2 gate (CDH5−/CD44−/CD43+), predicted to label the erythroid population, and P5 (CDH5+/CD44−/CD43+) exclusively generated erythroid colonies in a CFU assay (Fig. 3c). In contrast, cells in P3 (CDH5−/CD44+/CD43+) and P6 (CDH5+/CD44+/CD43+), predicted to label multipotent HPCs, were able to generate an array of colonies (Fig. 3c) including multilineage CFU-GEMM colonies at a higher rate when compared to unsorted cells.

In addition, we monitored the expression of CD34, CD43, CD44, and CDH5 by flow cytometry, from EHT D0 to EHT D3 (Additional file 1: Fig. S5). Our time course experiment showed that the described P1–P6 populations are generated over time starting from a population of CD34+/CD43−/CD44+low cells. Importantly, this initial population of cells at EHT D0 was devoid of any haematopoietic potential.

Although both CDH5+ (P6) and CDH5− (P3) cells displayed multilineage differentiation potential (as shown by CFU assays), immunofluorescence staining showed that the haematopoietic clusters contained CDH5+ but not CDH5− cells (Fig. 3a). CDH5− HPCs could only be detected when both the attached and floating fractions of cells were analysed by flow cytometry, indicating that they were floating in the culture medium and were most likely derived from CDH5+ HPCs. Thus, variable expression of CDH5 in HPCs might indicate different stages of their differentiation toward the haematopoietic lineage, with CDH5+ cells representing earlier progenitors and CDH5− HPCs being more mature progenitors that have detached from the endothelium. This is in agreement with previous studies showing that HSCs emerging in the AGM express CDH5, which is then downregulated upon HSC maturation [28].

In summary, these results validate the proposed cell identities and confirm their differentiation trajectories, demonstrating that endothelial cells are able to undergo EHT and endothelial-to-mesenchymal transition (EndoMT) [32–36] to generate haematopoietic and mesenchymal populations, respectively. Additionally, we identified a sorting strategy to enrich for HPCs generated in vitro and confirmed their multilineage haematopoietic potential using differentiation assays.

### Cell cycle progression is required for specification of HPCs

**Fig. 4:**
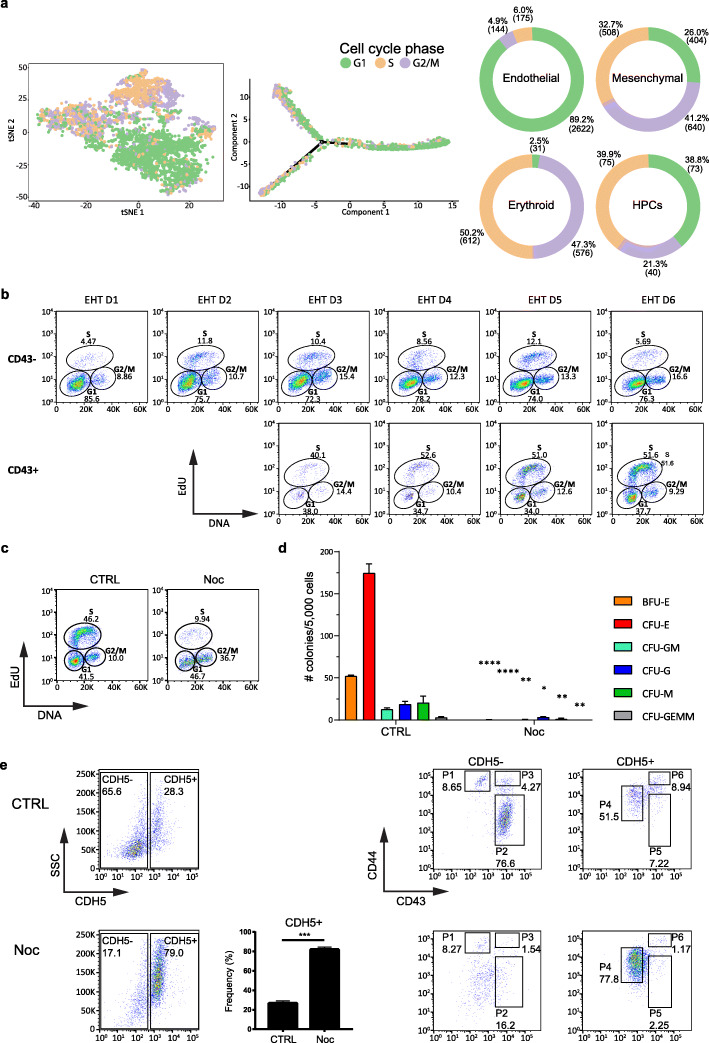
Cell cycle progression is necessary for EHT. **a** (Left panel) tSNE plot of the merged data from EHT D3 and EHT D5. Cells were coloured based on the cell cycle phase inferred from scRNA-seq data. (Middle panel) Cell cycle phase along differentiation trajectory, from endothelial cells (right) to haematopoietic and mesenchymal branches (left). (Right panel) Pie charts showing the percentage of cells in G1, G2/M, and S phase in each of the clusters. **b** EdU staining and flow cytometry were used to monitor the cell cycle profile of CD43− and CD43+ cells during differentiation. Results are representative of 3 independent experiments. **c**–**e** At EHT D3, media were supplemented either with 0.1% DMSO (CTRL) or with 0.1 μg/ml Nocodazole (Noc). After 48 h, at EHT D5, cells were either **c** processed for EdU staining and flow cytometry to monitor their cell cycle profile, **d** washed to remove Nocodazole and further cultured in a CFU assay to reveal their differentiation potential, or **e** stained for flow cytometry to monitor the distinct populations previously identified (P1, mesenchymal cells; P4, endothelial cells; P2 and P5, erythroid progenitors; P3 and P6, HPCs). Results are representative of 3 independent experiments. Data in **d** and bar plot in **e** are mean ± SEM of 3 independent experiments, with statistical significance compared to control as *P < 0.5, **P < 0.01, ***P < 0.001, and ****P < 0.0001 by two-tailed unpaired *t* test, and representative of 2 distinct hPSC lines. Degrees of freedom = 4; *t* values for the distinct colonies in **d** are BFU-E = 39.62, CFU-E = 15.74, CFU-GM = 6.871, CFU-G = 4.271, CFU-M = 4.867, and CFU-GEMM = 5.196; *t* value in **e** = 15.05

During the course of the experiments described above, we observed that clusters of HPCs contain cells with a distinct nuclear morphology compared to the surrounding endothelial and mesenchymal cells. Indeed, a majority of these cells displayed nuclear condensation typical of mitotic or dividing cells (Fig. 3a) thereby suggesting that HPCs represent a highly proliferative population. To confirm this observation, we used our scRNA-seq data to infer the cell cycle state of the HPC, endothelial, mesenchymal, and early erythroid populations (Fig. 4a) [26]. While endothelial cells were enriched in G1, mesenchymal cells and HPCs displayed a more active cell cycle. In contrast, erythroid progenitors appeared to be extremely proliferative with only 2.5% of the cells in G1 (Fig. 4a). In line with this observation, genes belonging to the Rb and CIP/KIP families such as *RB1*, *RBL2/p130*, and *CDKN1C/p57*, which inhibit G1 progression and G1/S transition, were gradually downregulated during the transition of endothelial cells to HPCs and upon their further differentiation to erythroid cells (Additional file 1: Fig. S6a). Concomitantly, proliferation markers such as *MKI67* and *CCNB2* were upregulated. Interestingly, we observed differential expression of cyclin D genes that are necessary for the activation of CDK4/6 and progression through the G1 phase. Endothelial cells were enriched for *cyclin D1* and *D2*, while HPCs appeared to switch to *cyclin D2* and *D3*. The more committed erythroid cluster preferentially expressed *cyclin D3* (Additional file 1: Fig. S6a). This might suggest a divergent requirement for cyclin D genes during EHT and would be in agreement with genetic studies in the mouse showing that the simultaneous absence of the three cyclin Ds leads to severe haematopoietic defects [37].

To experimentally validate these observations, we assessed the cell cycle profile of CD43+ (haematopoietic) and CD43− (endothelial and mesenchymal) cells at different time points during EHT using EdU incorporation assay (Fig. 4b). These experiments confirmed that the emerging CD43+ haematopoietic population was characterised by an active cell cycle profile, while the majority of CD43− cells were in the G1 phase. Furthermore, we took advantage of FUCCI-hPSCs [38, 39] to monitor changes in cell cycle phase during EHT (Additional file 1: Fig. S6c). We observed that emerging HPC clusters contained cells that mainly expressed the reporter specific for S/G2/M phases (GEMININ), while endothelial cells were found to be either positive for the late G1 reporter (CDT1) or negative for both reporters thus indicating G0/early G1 phase (Additional file 1: Fig. S6c). These findings support the notion that the endothelial differentiation into HPCs is associated with cell cycle entry.

We next asked whether RUNX1+ haemogenic endothelial cells present at EHT D3 could transit to haematopoietic progenitors if their cell cycle progression was blocked. To address this question, EHT D3 cells were grown for 48 h in the presence of Nocodazole, a small molecule commonly used to block cell cycle progression [40, 41]. The efficacy of Nocodazole treatment was confirmed by EdU incorporation analyses showing an expected enrichment of cells in G2/M (Fig. 4c). However, a significant fraction of cells remained in G1. These cells most likely represent a quiescent population of endothelial cells (Fig. 4a) that never entered cell cycle during the 48 h treatment. Following Nocodazole treatment, cells were washed to allow cell cycle progression and their haematopoietic potential was assessed by CFU assays. This showed that cell cycle inhibition effectively blocked the generation of functional haematopoietic cells (Fig. 4d). Consistently, flow cytometry analyses showed an enrichment in CDH5+ (endothelial) cells and depletion of the CD43+ (haematopoietic) compartment after Nocodazole treatment (Fig. 4e). Importantly, this effect was not related to non-specific cytotoxicity, as cells treated with Nocodazole only displayed a minor increase in cell death (Additional file 1: Fig. S6b).

**Fig. 5:**
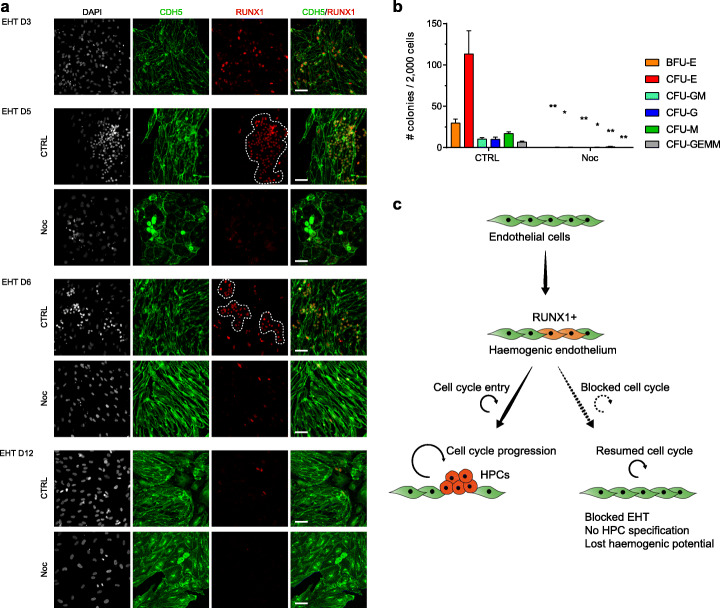
Endothelial cells lose haemogenic potential upon cell cycle block. **a** Immunofluorescence staining showing the expression of CDH5 and RUNX1 over time. At EHT D3, media were supplemented either with 0.1% DMSO (CTRL) or with 0.1 μg/ml Nocodazole (Noc). After 48 h, at EHT D5, cells were washed and media refreshed every other day. After cell cycle block is released, cells reprise proliferation, but RUNX1 expression is gradually lost, with positive cells representing very rare events at EHT D12. Haematopoietic clusters are outlined in CTRL and consist of RUNX1+ cells that are mainly round shaped and grouped in clusters. These clusters can also be recognised in the CDH5-stained panel (green), in which they display a typical round morphology, as opposed to the surrounding elongated endothelial cells. Finally, these clusters can also be recognised in the DAPI (grey) panel, in which they are characterised by the typical dense nuclear morphology and brighter appearance compared to endothelial cells. In Nocodazole-treated cultures, all these features are not present: the few RUNX1+ cells are more scattered and not grouped in clusters; they mark elongated endothelial cells rather than round cells; these elongated cells are CDH5 positive (green panel) and thus clearly endothelial; finally, their nuclei do not appear to be denser or brighter than the surrounding cells, as was the case in haematopoietic clusters found in control. Scale bar is 50 μm. **b** Forty-eight hours after Nocodazole release, at EHT D7, cells were collected and cultured in a CFU assay to assess their differentiation potential. Data are mean ± SEM of 3 independent experiments, with statistical significance compared to control as *P < 0.5, **P < 0.01, ***P < 0.001, and ****P < 0.0001 by two-tailed unpaired *t* test. Degrees of freedom = 4; *t* values for the distinct colonies are BFU-E = 5.803, CFU-E = 4.02, CFU-GM = 6.2, CFU-G = 3.625, CFU-M = 7.407, and CFU-GEMM = 5.547. **c** Diagram summarising the role of cell cycle progression during EHT

To confirm that blocking cell cycle does not affect the ability of endothelial cells to subsequently re-enter cell cycle and proliferate, but it rather influences their cell fate decision by precluding the haematopoietic fate choice, we performed release experiments. Following 48 h Nocodazole treatment from EHT D3 to EHT D5, cells were washed/released and further cultured for up to 1 week. Following release from cell cycle block, endothelial cells were able to re-enter cell cycle and resume proliferation, expanding as a monolayer, possibly composed of a mix of endothelial and mesenchymal cells. Importantly, immunofluorescence staining confirmed the progressive reduction in the number of RUNX1+ cells (Fig. 5a). The rare RUNX1+ cells detectable after treatment did not form distinct clusters; displayed an elongated endothelial morphology, as assessed by immunofluorescence and light microscopy; and were unable to acquire colony forming potential (Fig. 5b). In line with these results, there were no visible HPC clusters even after one additional week of culture.

Taken together, these experiments confirmed that blocking cell cycle prevents transition of haemogenic endothelial cells into haematopoietic progenitors (Fig. 5c). Furthermore, when cell cycle is blocked, haemogenic endothelial cells progressively lose the expression of RUNX1 and adopt a non-haemogenic endothelial identity or possibly assume a mesenchymal fate.

### Inhibition of specific cyclin-dependent kinase (CDK) proteins impairs EHT

To further explore the connection between cell cycle regulation and haematopoietic differentiation, we focused on CDK proteins and their role during EHT. CDK proteins are key regulators of cell cycle progression and are involved in a variety of non-canonical functions such as transcriptional activation, DNA damage repair, metabolism, differentiation, and cell fate decision in multiple systems [39, 42–45]. To this end, we used three well-characterised inhibitors which bind to CDK proteins and block their phosphorylation activity (PD0332991 for CDK4 and CDK6, RO3306 for CDK1, and Roscovitine for CDK2 and to a minor extent CDK1) [46–53].

**Fig. 6:**
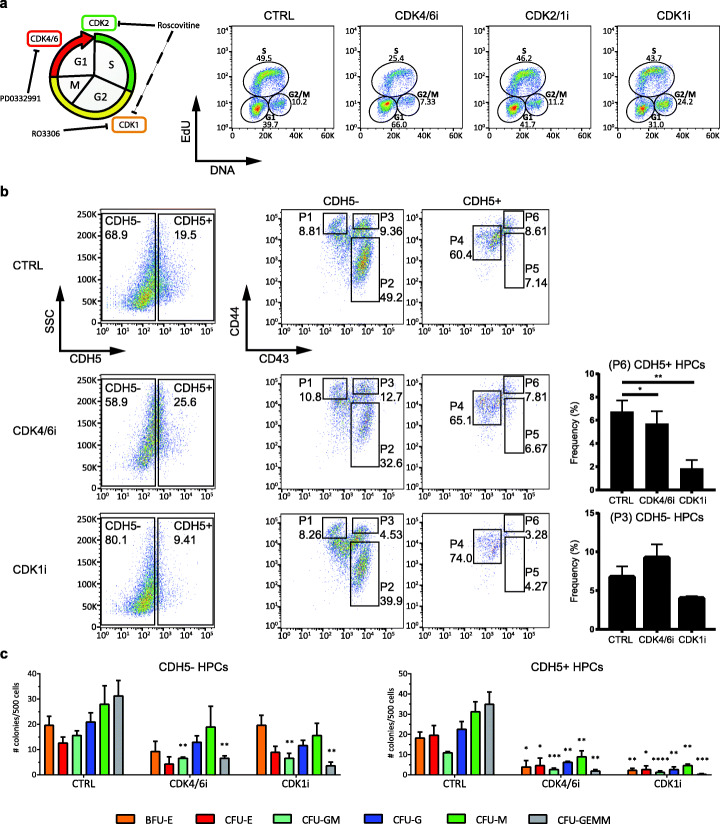
Functional effect of CDK inhibitors during EHT. **a** Schematic showing inhibitors used in the experiments (left panel) and their effect on cell cycle progression (right panel). Samples were treated for 48 h between EHT D3 and EHT D5 with either 0.1% DMSO (CTRL), 1 μM PD0332991 (CDK4/6i), 10 μM RO3306 (CDK1i), or 4 μM Roscovitine (CDK2/1i). At EHT D5, cells were either **a** processed for EdU staining and flow cytometry to monitor cell cycle profile or **b** stained for flow cytometry to monitor the frequency of distinct cell populations generated (P1, mesenchymal cells; P4, endothelial cells; P2 and P5, erythroid progenitors; P3 and P6, HPCs). Results are representative of 3 independent experiments. **c** At EHT D5, cells were washed to remove the inhibitors and two populations of HPCs (corresponding to P3 and P6 in **b**) were FACS-sorted and further cultured in a CFU assay to reveal their differentiation potential. Data are mean ± SEM of 3 independent experiments, with statistical significance compared to control as *P < 0.5, **P < 0.01, ***P < 0.001, and ****P < 0.0001 by one-way ANOVA. Degrees of freedom in **b** for CDH5− HPCs are DFn = 1.103, DFd = 2.205, *F* value 11.19, and for CDH5+ HPCs are DFn = 1.046, DFd = 2.092, *F* value 204.2. Degrees of freedom in **c** are DFn = 2, DFd = 6; *F* values for the distinct colonies in CDH5− HPCs are BFU-E = 2.483, CFU-E = 2.771, CFU-GM = 12.15, CFU-G = 3.261, CFU-M = 0.8633, and CFU-GEMM = 17.43; *F* values for the distinct colonies in CDH5+ HPCs are BFU-E = 12.51, CFU-E = 6.757, CFU-GM = 67.36, CFU-G = 21.71, CFU-M = 19.26, and CFU-GEMM = 30.63

In short, EHT D3 cells were grown in the presence of each inhibitor for 48 h and were washed and collected at EHT D5 for further analyses. Specifically, we assessed the cell cycle profile of unenriched populations by EdU staining and the colony forming capacity of sorted HPCs (Fig. 6). Consistent with the known functional redundancy between CDKs, the treatments were not able to completely block cell cycle but instead induced enrichment in specific phases. Thus, this approach was fundamentally different compared to the use of Nocodazole, as it allowed us to examine the role of distinct cell cycle regulators rather than cell cycle progression. More precisely, inhibition of CDK4/6 (CDK4/6i) increased the number of cells in G1, Roscovitine (CDK2/1i) did not significantly change cell cycle profile, and inhibition of CDK1 (CDK1i) caused an increase in the number of cells in G2/M (Fig. 6a).

Further analysis revealed that CDK4/6i treatment resulted in a reduction in the number of CD43+ cells and a modest effect on the frequency of HPCs (Fig. 6b). However, the differentiation potential of both CDH5+ and CDH5− HPCs was disrupted since their capacity to form blood colonies was significantly reduced (Fig. 6c). Roscovitine was not associated with major modifications in the frequency of populations generated during EHT. Finally, CDK1i significantly decreased the number of HPCs and to a minor extent the number of erythroid CD43+ cells (Fig. 6b). The smaller fraction of HPCs produced displayed a strong impairment in their differentiation potential as shown by CFU assays (Fig. 6c). Similarly, the inducible knockdown of CDK1 depleted the colony forming potential of the EHT culture, thus confirming our results using a genetic approach (Additional file 1: Fig. S7).

Taken together, these experiments suggest that specific CDKs are necessary not only for EHT to occur but also for HPCs to establish their full differentiation potential. Importantly, the inhibition of distinct cell cycle regulators, as opposed to cell cycle block, alters, rather than inhibit, the EHT process leading to HPC specification.

### Inhibition of CDK regulators disrupts EHT

**Fig. 7:**
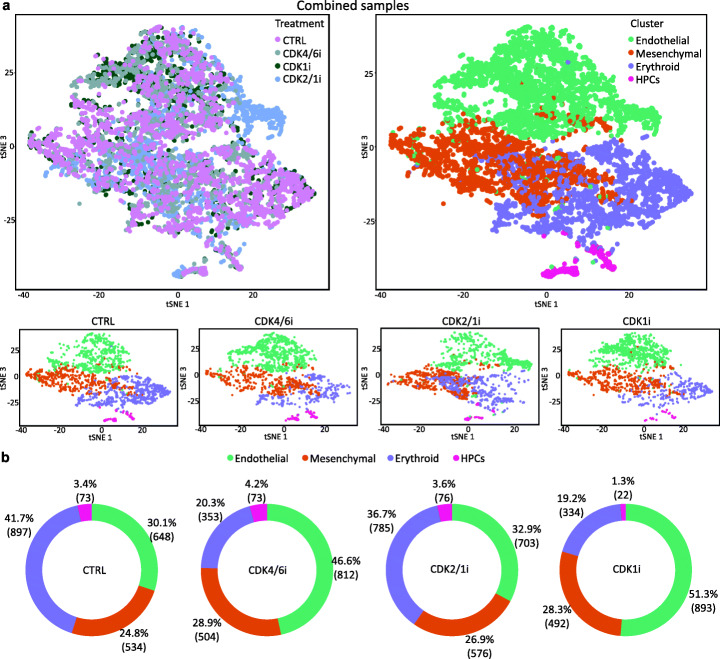
Effect of CDK inhibitors evaluated by single-cell transcriptomics. Samples were treated with different cell cycle inhibitors, namely PD0332991 (CDK4/6i), Roscovitine (CDK2/1i), and R03306 (CDK1i), or with DMSO (CTRL). At EHT D5, cells were collected and processed for scRNA-seq. Data from the four samples was merged and clustered. **a** tSNE plots showing previously identified cell populations, on the merged samples (top row) and for each treatment separately (bottom row). **b** Pie charts showing the percentage and absolute number of cells for each condition

In order to further understand the effect of CDK inhibitors on the EHT process and the differentiation capacity of HPCs, we performed scRNA-seq analyses on EHT D5 cells grown in the presence of each CDK inhibitor for 48 h. Following quality control, we analysed 1742 cells treated with CDK4/6i, 2140 with CDK2/1i, and 1741 with CDK1i. As expected, in each of the samples, we identified HPC, endothelial, mesenchymal, and erythroid populations (Fig. 7a).

Comparative analysis showed increase in the percentage of endothelial cells upon inhibition of CDK4/6, associated with a decrease in the erythroid compartment. Endothelial cells are mostly non-cycling cells (Fig. 4a, b, Additional file 1: Fig. S6a, c), and in control conditions, their numbers decrease during EHT concomitantly with production of haematopoietic cells (Fig. 2b). Thus, our data suggest that the increase in endothelial cells following CDK4/6 inhibition, compared to control, is not associated with proliferation but rather with their decreased transition to the haematopoietic fate, i.e. decreased EHT (Fig. 7b). The percentage of HPCs in the presence of the inhibitor was comparable to control, confirming our flow cytometry data (Fig. 6b) However, these HPCs displayed impaired differentiation potential (Fig. 6c). This could explain the reduction in the erythroid compartment, and why the frequency of HPCs remained comparable to control despite reduced EHT.

Cells grown in the presence of the CDK1i also displayed increase in the percentage of endothelial cells and reduction in the erythroid cluster, suggesting a reduced progression through EHT. However, the number of HPCs was strongly reduced when compared to control, again in agreement with our previous flow cytometry analyses (Fig. 6b). This indicates a key role for CDK1 activity in the HPC population.

**Fig. 8:**
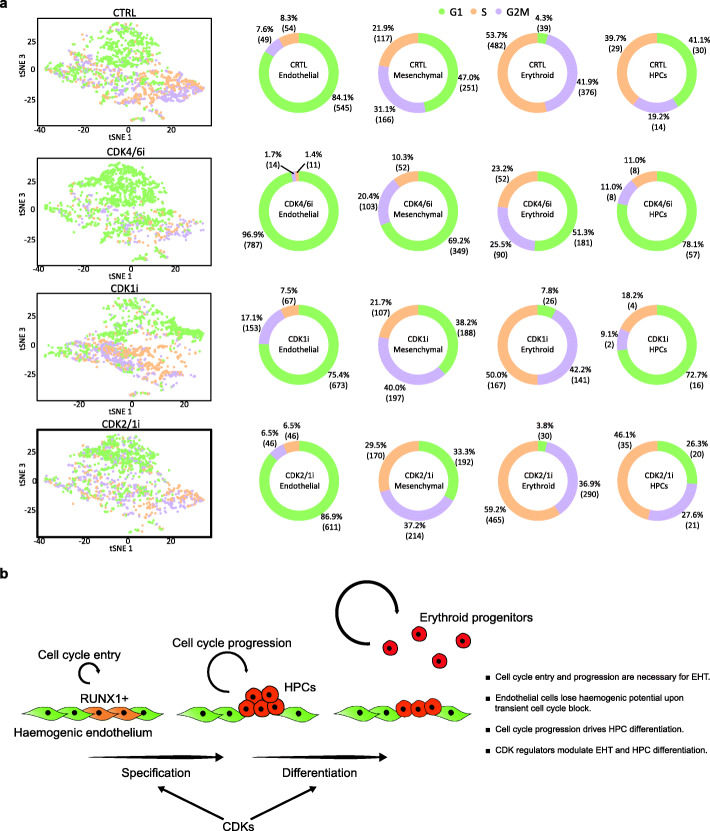
Cell cycle regulation controls EHT. **a** Cell cycle marker genes identified in the scRNA-seq dataset were used to determine the cell cycle profile of individual clusters upon inhibition of distinct CDKs. **b** Proposed model for the role of cell cycle during EHT. Endothelial cells need to re-enter the cell cycle in order to progress through EHT and generate functional HPCs. Additionally, endothelial cells lose their haemogenic potential if cell cycle entry and progression is prevented upon transient cell cycle block. The process is regulated by CDKs, which are necessary for EHT and for the acquisition of HPC differentiation potential

Next, we inferred the cell cycle state by transcriptome analysis of cells in each of the clusters across all treatments. Inhibition of CDK4/6 caused an expected enrichment in G1 of all four populations (Fig. 8a). On the other hand, the inhibition of CDK1 was associated with the expected enrichment in G2/M in endothelial and mesenchymal clusters (Fig. 8a). Endothelial cells blocked in this phase of the cell cycle most probably represent those that were undergoing either EHT or EndoMT at the start of the treatment. Indeed, while the majority of endothelial cells are in G1, those that are transitioning to haematopoietic and mesenchymal fates are associated with re-entry into the cell cycle and display downregulation of cell cycle inhibitors concomitant with upregulation of proliferation markers (Fig. 4a, b, Additional file 1: Fig. S3). Despite the presence of the CDK1 inhibitor, few HPCs were found in S/G2/M, indicating that the treatment either caused HPCs to further differentiate to the erythroid compartment or it affected their survival. Either options (i.e. differentiation to erythroid progenitors or cell death of HPCs upon CDK1 inhibition) could account for the decrease in the HPC cluster compared to pre-treatment levels and for the fact that the remaining HPCs were mostly in G1 phase, i.e. they were not cycling at the time of the treatment and were not affected by the inhibition of CDK1 which gets activated in G2/M. Importantly, the few remaining progenitors, enriched in G1, also displayed a depleted differentiation potential, similarly to HPCs enriched in G1 by the CDK4/6i treatment (Fig. 6c). This suggests that the timely activation of these cell cycle regulators is required for the acquisition of haematopoietic differentiation potential.

Overall, along with further confirming its requirement for EHT, these results show that cell cycle regulation controls HPC differentiation. Additionally, the two regulators possibly play distinct roles in HPCs. CDK4/6 might be necessary for the acquisition of differentiation potential, as its inhibition preserved the HPC population although negatively affecting its ability to differentiate. CDK1 might be more important for maintaining the HPC population, as the population was eroded when CDK1 was inhibited and only non-cycling cells were able to preserve the HPC state.

Of note, inhibition of either CDK4/6 or CDK1 resulted in modest but broad transcriptional changes across all cell populations compared to control. We mainly observed downregulation of genes for ribosomal subunits and enrichment in GO terms involved in mitochondrial functioning (Additional file 1: Fig. S8, Additional file 2: Tables S1 – S8). Furthermore, HPCs showed upregulation of genes such as *ALAS2* or haemoglobin genes, suggesting their priming or precocious differentiation toward the erythroid lineage. Importantly, the inhibition of CDK1 was able to affect the transcriptional signature of the HPC population despite not affecting its cell cycle profile, confirming that this regulator may play a role during EHT beyond cell cycle regulation. Unlike CDK4/6 and CDK1, the CDK2/1 inhibitor did not cause major transcriptional changes compared to control, consistent with the fact that this treatment did not affect the EHT process.

To conclude, our single-cell analyses indicate that the inhibition of CDK4/6 and CDK1 was able to cause transcriptional changes that were not strictly related to cell cycle progression. Despite the modest extent of these changes, our findings suggest that these CDKs can participate in a complex crosstalk between cell cycle regulation and the transcriptional programmes that drive EHT.

### In vitro EHT culture recapitulates populations found in the human AGM in vivo

In order to assess the relevance of our findings for human foetal blood development, we compared the cells generated in our study with a publicly available dataset obtained from human AGM [54]. Having annotated this data according to the Authors’ original cell annotations, we then mapped this dataset onto our data. This allowed us to obtain predictions of the cell identities in our data using the annotations from the in vivo generated dataset (Additional file 1: Fig. S10a). This approach confirmed that endothelial, mesenchymal, and haematopoietic cells identified in our study displayed similarity with corresponding populations described in vivo*.*

Furthermore, endothelial cells generated in our system expressed top marker genes of both haemogenic and arterial endothelium from AGM (Additional file 1: Fig. S10b). Based on the level of expression of marker genes, in vitro generated endothelial cells displayed higher similarity to late haemogenic endothelial cells (late HECs), which give rise to true HSPCs in the AGM [54], rather than early haemogenic endothelial cells (early HECs). Interestingly, in vitro derived HPCs displayed similarities to end-stage HSPCs from AGM, albeit maintaining expression of genes expressed in immature HSPCs such as active cell cycle genes (Additional file 1: Fig. S10b).

Overall, our comparative analysis showed that in vitro generated endothelial and haematopoietic cells share many similarities to populations found in the human AGM and thus make a valuable model system for studying EHT.

## Discussion

In this study, we have uncovered a link between cell cycle regulation and endothelial-to-haematopoietic transition (Fig. 8b). By combining a hPSC differentiation system with single-cell transcriptomics, we gained insights into the mechanisms driving early human haematopoietic development.

Our data suggest that the function of cell cycle regulators goes beyond their canonical role in proliferation (Additional file 1: Fig. S9). Thus, CDK4/6 appears to be necessary for both specification of HPCs (i.e. EHT) and their further differentiation. In agreement with our results, genetic studies in the mouse have shown that the cyclin D-CDK4/6 complex is indeed required for haematopoietic development [37, 55]. CDK1 also appears to be necessary for EHT and for balanced HPC differentiation and survival. Of note, CDKs are known to be involved in a variety of non-canonical functions such as transcriptional control and cell fate decision [39, 42–45, 56]. Accordingly, our results showed that the activation of CDK4/6 and CDK1 is required during EHT. Their inhibition was able to affect the transcriptional signature of the distinct populations generated in culture as well as the differentiation capacity of nascent HPCs, further suggesting that the role of these regulators during EHT is not strictly related to cell cycle progression.

Based on these results, we propose a tight link between cell cycle regulation and EHT (Fig. 8b). Our model suggests that haemogenic endothelial cells are a population of quiescent or slow proliferating cells. Their transition to the haematopoietic fate is associated with cell cycle re-entry and the acquisition of an active cell cycle profile. This ultimately results in the specification of multipotent HPCs, a transitory population of cells which in the current culture conditions quickly commit to highly proliferative erythroid progenitors. We have shown that cell cycle progression is necessary for EHT and that upon blocking cell cycle, haemogenic endothelial cells are unable to transition to the haematopoietic fate. As a result, they downregulate RUNX1, lose haemogenic potential, and instead adopt a non-haemogenic endothelial cell identity, even when the cell cycle block is removed (Fig. 5c, Additional file 1: Fig. S9a). This would suggest that EHT can occur within a narrow time window during which cell cycle entry and progression are necessary for the completion of the haemogenic programme. This is possibly modulated by the timely activation of specific cell cycle regulators.

Recent studies in the mouse have demonstrated that the generation of early haematopoietic progenitors in the AGM is associated with a dynamic cell cycle regulation [57]. Our analysis here goes beyond these initial findings and shows that the endothelial cells express key cell cycle inhibitors of the Rb and CIP/KIP families, such as *RB1*, *p21*, and *p57* (Additional file 1: Fig. S3a). The downregulation of these inhibitors and concomitant expression of key haematopoietic markers in a subset of endothelial cells mark their re-entry into the cell cycle and the onset of haematopoietic specification. Importantly, blocking cell cycle progression effectively prevents this transition, suggesting that cell cycle could be necessary to orchestrate the expression of key lineage regulators and thus differentiation mechanisms.

Interestingly, our group has previously shown that hPSCs acquire responsiveness to distinct differentiation signals during specific phases of the cell cycle, through a post-translational modulation of SMAD2/3 transcriptional activity controlled by cyclin D-CDK4/6 [39]. We hypothesise that a similar regulation could occur during EHT and that CDKs could be required for the acquisition of functional haematopoietic identity. Accordingly, it was previously reported that CDK6 can bind to RUNX1 and interfere with its transcriptional activity [58]. RUNX1 is an essential regulator of EHT, which synergises with other key factors such as TAL1 and GATA2, and is necessary for the induction of the haematopoietic transcriptional programme. Thus, it is possible that an important role for the cell cycle machinery is to modulate the timely activation of the haematopoietic transcriptional network. Importantly, our results show that when this modulation is impeded by preventing cell cycle progression and disrupting the activation of the cell cycle machinery, endothelial cells permanently lose their haemogenic identity. Our data also imply that EndoMT and EHT represent two alternative cell fate choices undertaken by endothelial cells during foetal haematopoietic development. The mesenchymal population does not appear to be affected by the perturbations in the cell cycle used in this study. However, commitment toward mesenchymal cells appears to take place at an earlier stage, at EHT D0 or earlier, and to be similarly associated with the expression of cell cycle related genes (Additional file 1: Fig. S3b). This would suggest that despite EndoMT and EHT taking place at slightly different time windows, endothelial cells similarly need to enter and progress through cell cycle in order to differentiate toward both haematopoietic and mesenchymal fates. Further investigations would allow to explore this possibility and to assess whether blocking cell cycle at an earlier time point might be able to prevent EndoMT and maximise the number of cells available for EHT once this block is lifted.

In conclusion, our study has demonstrated a complex interplay between the molecular machineries that control cell cycle progression and EHT. This is achieved through the activation of specific cell cycle regulators, which define the transcriptional landscape and control the differentiation potential of populations generated during EHT. Further understanding the mechanisms involved in the specification of HSPCs will help to design more effective culture conditions for their ex vivo expansion and pave the way to the effective in vitro generation of HSPCs with long-term engraftment potential.

## Methods

### Culture of hPSCs

Cell lines used in this study were previously authenticated.

The hIPSC lines A1AT-RR [59] and FSPS13B were maintained as previously described [60] on plates coated with 10 μg/ml Vitronectin (STEMCELL Technologies) and cultured in E6 media supplemented with 2 ng/ml TGFβ (R&D) and 25 ng/ml FGF2 (Dr. Marko Hyvönen, Department of Biochemistry, University of Cambridge). Cells were maintained at 37 °C and 5% CO_2_, and passaged every 5–6 days by dissociation with 0.5 mM EDTA (ThermoFisher Scientific). For coating plates, 10 μg/ml Vitronectin in PBS (ThermoFisher Scientific) was applied for at least 1 h at room temperature.

### Haematopoietic differentiation

Differentiation was performed at 5% O_2_ by adapting a protocol previously described [23]. Briefly, a serum-free differentiation (SFD) medium was used, consisting of 75% Iscove’s modified Dulbecco’s medium (ThermoFisher Scientific) and 25% Ham’s F12 medium (ThermoFisher Scientific) supplemented with 1% N2 (ThermoFisher Scientific), 0.5% B27 (ThermoFisher Scientific), 0.05% BSA (Sigma-Aldrich), 1 mM ascorbic acid (2-phospho-l-ascorbic acid trisodium salt, Sigma-Aldrich), 4.5 × 10^−4^ monothioglycerol (Sigma-Aldrich), 2 mM l-glutamine (ThermoFisher Scientific), 150 μg/ml transferrin (Sigma-Aldrich), and 10 ng/ml penicillin/streptomycin (ThermoFisher Scientific). Undifferentiated cells were dissociated using 0.5 mM EDTA, and small aggregates were resuspended in SFD medium supplemented with 10 ng/ml BMP4 (R&D) and cultured as embryoid bodies (EBs) on non-treated plates (Starlab). After 24 h, an equivalent volume of SFD was added directly on top to not perturb the small EBs, supplemented with final concentrations of 10 ng/ml BMP4 and 5 ng/ml FGF2. At day 2, developing EBs were collected and washed. For this, they were centrifuged 3 min at 100*g*; gently resuspended in SFD supplemented with 10 ng/ml BMP4, 5 ng/ml FGF2, and 3 μM CHIR99021 (Tocris); and seeded back on the plate. After 24 h, EBs were again collected and washed, this time by centrifuging at 300*g* for 3 min, and resuspended in StemPro-34 SFM (ThermoFisher Scientific) supplemented with 1 mM ascorbic acid, 4.5 × 10^−4^ monothioglycerol, 2 mM l-glutamine, 150 μg/ml transferrin, 10 ng/ml penicillin/streptomycin (from now on referred to as complete SP34) containing 5 ng/ml FGF2, 15 ng/ml VEGF (Peprotech), 10 ng/ml IL-6 (R&D), and 5 ng/ml IL-11 (R&D), and cultured for 48 h. At day 5, EBs were again collected, washed, and resuspended in complete SP34 with 5 ng/ml FGF2, 15 ng/ml VEGF, 10 ng/ml IL-6, 5 ng/ml IL-11, 50 ng/ml SCF (R&D), 5 ng/ml IGF-1 (R&D), and 2 U/ml EPO (R&D) and cultured until day 8. At this stage, EBs were collected and prepared for sorting. For this, they were incubated for 20 min at 37 °C with collagenase solution, composed of Advanced DMEM/F12 (ThermoFisher Scientific), 20% KSR (ThermoFisher Scientific), 1% l-glutamine, and 1 mg/ml collagenase IV (ThermoFisher Scientific), followed by 3 min incubation with TrypLE (ThermoFisher Scientific). Single cells were then stained for flow cytometry, and the CD34+/CD43− fraction was sorted and used for the second stage of differentiation. For this, the population was resuspended at a density of 10^6^ cells/ml in complete SP34 with 10 ng/ml BMP4, 5 ng/ml FGF2, 5 ng/ml VEGF, 10 ng/ml IL-6, 5 ng/ml IL-11, 100 ng/ml SCF, 25 ng/ml IGF-1, 30 ng/ml TPO (Peprotech), 30 ng/ml IL-3 (Peprotech), 10 ng/ml Flt3-L (R&D), 20 ng/ml SHH (R&D), 10 ng/ml angiotensin II (Sigma-Aldritch), and 100 μM losartan potassium (R&D). Cells were transferred to a non-treated round-bottom 96-well plate (Corning), 200 μl/well (corresponding to 2 × 10^5^ cells/well); centrifuged 3 min at 300*g*; and incubated overnight to allow the cells to re-aggregate. On the following day, marking EHT D1, the small aggregates were plated on Matrigel (Corning). For this, they were gently transferred to thin-layer Matrigel-coated wells, with a density of 2 × 10^5^ cells/well in a 24-well plate, and cultured for additional 2–4 days using the same media, replaced every 2 days. For coating plates, Matrigel was diluted into cold medium with a concentration of 35 μg/cm^2^ of surface to be coated, and applied overnight at 37 °C. For treatments with cell cycle inhibitors, 0.1 μg/ml Nocodazole (Sigma-Aldrich), 1 μM PD0332991 (Tocris), 4 μM Roscovitine (Sigma-Aldrich), 10 μM RO3306 (Sigma-Aldrich), or 0.1% DMSO were added at EHT D3 for 48 h.

### Isolation of CD34+ peripheral blood mononuclear cells

Under sterile conditions, peripheral blood was diluted with room temperature PBS supplemented with 1 M trisodium citrate and 20% human serum albumin. Two volumes of blood dilution were transferred in 50-ml tubes on a layer of 1 volume of Ficoll-Paque (Sigma-Aldrich). After centrifugation for 15 min at 800*g*, the resulting layer of mononuclear cells was carefully removed, transferred to a new tube, further diluted using the same buffer, and centrifuged for 6 min at 600*g*. The resulting supernatant was removed; the pellet was resuspended in cold PBS supplemented with 0.5 M EDTA and 20% human serum albumin, and centrifuged again for 6 min at 600*g*. Cells were then processed for CD34+ enrichment using labelling with magnetic beads. Briefly, cells were resuspended in the same PBS/EDTA/human serum albumin buffer, added with 50 μl/10^8^ cells of FcR blocking reagent (Miltenyi Biotec) and 50 μl/10^8^ cells of CD34 magnetic beads (Miltenyi Biotec), and incubated for 30 min at 4 °C. After incubation, cells were washed using the same buffer and processed with AutoMACS Pro Separator to enrich for CD34+ peripheral blood mononuclear cells.

### May-Grünwald-Giemsa staining

Cells were resuspended in culture media and concentrated by cytospin centrifugation at 700*g* for 5 min onto SuperFrostPlus slides (ThermoFisher Scientific) using a Shandon Cytospin 3 cytocentrifuge. Slides were fixed for 3 min in cold methanol and stained with May-Grünwald-Giemsa (Sigma). Images were captured using a Leica DM5000b microscope in conjunction with a × 63 oil-immersion lens and an Olympus DP72 camera.

### Flow cytometry

Cells were dissociated into single cells using TrypLE for 3 min at 37 °C, washed with 0.1% BSA-PBS, and either fixed with 1% paraformaldehyde and kept at 4 °C for maximum 1 week or immediately stained for flow cytometry. For the staining, after a wash with PBS, cells were blocked with 10% donkey serum (Bio-Rad) for 30 min at room temperature. Cells were then stained with the relevant conjugated antibodies diluted in PBS for 1 h at room temperature, protected from light (Table 1). Following two washes with PBS, cells were analysed using the Cyan ADP flow cytometer or sorted on the BD Influx cell sorter. Data was analysed using the FlowJo VX software.

**Table 1:** Antibodies used for flow cytometry

Target	Supplier	Clone
CD34	Biolegend	581
CD43	BD Biosciences	1G10
CDH5	Biolegend	BV9
CD44	Biolegend	BJ18
CD90	Biolegend	5E10
CD31	Biolegend	WM59

Cell viability upon Nocodazole treatment was assessed using the Dead Cell Apoptosis Kit with Annexin V Alexa Fluor 488 & Propidium Iodide (ThermoFisher Scientific).

### RNA extraction, cDNA synthesis, and qPCR

Total RNA was extracted using the GenElute Mammalian Total RNA Miniprep Kit (Sigma-Aldrich) and the On-Column DNase I Digestion set (Sigma-Aldrich) according to the manufacturer’s instructions. RNA was reverse-transcribed using 250 ng random primers (Promega), 0.5 mM dNTPs (Promega), 20 U RNAseOUT (Invitrogen), 0.01 M DTT (Invitrogen), and 25 U of SuperScript II (Invitrogen). For the qPCR reaction, the resulting cDNA was diluted 30-fold. Quantitative PCR mixtures were prepared using the KAPA SYBR FAST qPCR Master Mix Kit (Kapa Biosystems), 4.2 μl of cDNA, and 200 nM of each of the forward and reverse primers (Table 2). Technical duplicates of the samples were run on 384-well plates using the QuantStudio 12 K Flex Real-Time PCR System machine, and results analysed using the delta cycle threshold (ΔCt) method. Expression values were normalised to the housekeeping gene RPLP0.

**Table 2:** Primers used for qPCR

Gene	Forward primer (5′–3′)	Reverse primer (5′–3′)
*CDH5*	TGGCCAGCTGGTCCTGCAGAT	TGCCCGTGCGACTTGGCATC
*CDK1*	GGAAGGGGTTCCTAGTACTGC	AAGCACATCCTGAAGACTGACT
*CDK2*	CTCCAGGGCCTAGCTTTCTG	CCGGCGAGTCACCTCATGG
*CDK4*	TGAGGGGGCCTCTCTAGCTT	CAAGGGAGACCCTCACGCC
*CDK6*	ACAGAGCACCCGAAGTCTTG	GGGAGTCCAATCACGTCCAA
*CDKN1A/p21*	GGCAGACCAGCATGACAGAT	GATGTAGAGCGGGCCTTTGA
*CDKN1B/p27*	TAATTGGGGCTCCGGCTAAC	GAAGAATCGTCGGTTGCAGGT
*CDKN1c/p57*	GCTGCGGTGAGCCAATTTAG	AACAAAACCGAACGCTGCTC
*CYCLIN B1*	CGCCTGAGCCTATTTTGGTTG	AGTGACTTCCCGACCCAGTA
*CYCLIN D1*	GCTGTGCATCTACACCGACA	AAATGAACTTCACATCTGTGGCA
*CYCLIN D2*	GCCACCGACTTTAAGTTTGC	CGGTACTGCTGCAGGCTATT
*CYCLIN D3*	TGTGCTACAGATTATACCTTTGCC	GCTTCGATCTGCTCCTGACA
*CYCLIN E1*	GACGGGGAGCTCAAAACTGA	TCGGGCTTTGTCCAGCAAAT
*GATA1*	CTACACCAGGTGAACCGGC	CTTTTCCAGATGCCTTGCGG
*GATA2*	CTGTTCAGAAGGCCGGGAG	AATTTGCACAACAGGTGCCG
*GFI1*	ATCCACACTGGTGAGAAGCC	GCTGCCCTCTGTAGTGTTGT
*LMO2*	CAGAACATTGGGGACCGCTA	GTCTTGCCCAAAAAGCCTGA
*KLF1*	CCGAGGAAGAGGAGGCTTGAG	GGAAGTCATCCTGTGTGTCCG
*MEIS1*	GCGCAAAGGTACGACGATCT	GGTACTGATGCGAGTGCAGA
*MYB*	GCTACTGCCTGGACGAACTG	GTTGTTAACAGTGGGCTGGC
*PECAM1*	CAGGCGCCGGGAGAAGTGAC	CGTCCAGTCCGGCAGGCTCT
*RB1*	CTGTGGATGGAGTATTGGGAGG	TCTCATCTAGGTCAACTGCTGC
*RBL1/p107*	AGCAGAGGAGGATTCCTTGCAG	GGGCACATAATCGCATTGGC
*RBL2/p130*	GCTACACGCTGGAGGGAAAT	TCCTTCCACTGTCCCTTTGC
*RPLP0*	GGCGTCCTCGTGGAAGTGAC	GCCTTGCGCATCATGGTGTT
*RUNX1*	CATCGCTTTCAAGGTGGTGG	CATGGCTGCGGTAGCATTTC
*SPI1*	CCCCACGACCGTCCAG	GTAATGGTCGCTATGGCTCTCC
*TAL1*	TACTGATGGTCCCCACACCA	CCAGGCGGAGGATCTCATTC
*VWF*	TTACGTGGGTGGGAACATGG	TCTGTGGTGACTGTGCCATC

### CFU assay

The assay was performed using the MethoCult H4435 Enriched medium (STEMCELL Technologies), following the manufacturer’s instructions. Briefly, cells were added in a tube with the medium, mixed by vortex, plated on non-treated 35-mm culture dishes (Corning), and incubated for 14 days at 37 °C, 5% CO_2_ and 5% O_2_. Different types of colony were recognised based on morphology, counted, and, when relevant, collected for RNA extraction.

### Cell cycle profile analysis

Cell cycle profile analysis was performed using the Click-iT EdU Alexa Fluor 488 Flow Cytometry Assay Kit (ThermoFisher Scientific) according to the manufacturer’s instructions. Briefly, cultured cells were incubated at 37 °C with 10 μM EdU for 1 h and harvested after dissociation with TrypLE. After 3 washes with 0.1% BSA-PBS, cells were fixed with 1% paraformaldehyde for 15 min at room temperature and washed three more times with 0.1% BSA-PBS. Cells were then permeabilised for 15 min with saponin-based permeabilisation/wash buffer and incubated with the Click-iT reaction cocktail for 30 min protected from light. Cells were washed once with saponin-based permeabilisation/wash buffer, stained for DNA content using DAPI (ThermoFisher Scientific), and analysed on the Cyan ADP flow cytometer and FlowJo VX software.

### Immunofluorescence staining

Cells were fixed with 4% paraformaldehyde and kept at 4 °C for maximum 1 week or immediately stained for immunohistochemistry. Cells were blocked/permeabilised with 1% Donkey serum in PBS + 0.3% Triton X-100 for 1 h, and then incubated with the relevant primary antibodies diluted in PBS + 0.03% Triton X-100 at 4 °C overnight (Table 3). Cells were then washed three times with PBS and incubated with secondary antibodies diluted in PBS + 0.03% Triton X-100 at 4 °C for 1 h.

**Table 3:** Antibodies used for immunostaining

Target	Supplier	Catalogue number
RUNX1	Biolegend	659302
CD43	BD Biosciences	560198
CDH5	R&D	AF938
CD44	Abcam	Ab157107
VIMENTIN	Dako	M7020

### Inducible knockdown

Plasmid carrying an inducible shRNA was generated as previously described [61]. Briefly, shRNA for the CDK1 gene (shCDK1) was obtained from previous publication [62] and introduced by cloning of annealed oligonucleotides in the sOPTiKD plasmid between the BglII and SalI-HF sites (Table 4).

**Table 4:** Oligonucleotides for shCDK1

Oligo	Sequence
Top	GATCCCGCTGTACTTCGTCTTCTAATTCTCGAGAATTAGAAGACGAAGTACAGCTTTTTTG
Bottom	TCGACAAAAAAGCTGTACTTCGTCTTCTAATTCTCGAGAATTAGAAGACGAAGTACAGCGG

The resulting sOPTiKD_shCDK vector was targeted to the AAVS1 locus in combination with the pZFN.AAVS1-KKR and pZFN.AAVS1-ELD vectors [61]. Single-cell hPSCs were nucleofected using the Lonza P3 Primary Cell 4D-Nucleofector X Kit and the cycle CA-137 on a Lonza 4D-Nucleofector System, in feeder-free conditions. Monoclonal colonies were selected for 7–10 days with 1 μg/ml puromycin (Sigma). Expression of the shCDK1 was induced during differentiation by adding 1 μg/ml tetracycline hydrochloride (Sigma-Aldrich) at EHT D2. At EHT D5, the efficiency of knockdown was determined by qPCR, and haematopoietic potential for total culture was assessed by CFU assay.

### Statistical analysis

Statistical analyses were performed using the GraphPad Prism 7 software. The type of statistical analysis performed and the number of replicates used in each experiment are described in the figure legends. For the comparison of two or multiple groups, two-tailed unpaired *t* test or one-way ANOVA test was performed, respectively. Significance in each analysis is represented as *P < 0.5, **P < 0.01, ***P < 0.001, and ****P < 0.0001.

### Single-cell RNA sequencing

Following dissociation, cells were resuspended at a concentration of 1500 cells/μl in ice-cold SP34 medium, complete with cytokines as for EHT culture. Libraries were constructed using Chromium Controller and Chromium Single Cell 3′ Library & Gel Bead Kit (10x Genomics) according to the manufacturer’s instructions for the recovery of 2000 cells for each sample. Briefly, the cellular suspension was added to the master mix containing nuclease-free water, RT Reagent Mix, RT Primer, Additive A, and RT Enzyme Mix. Master mix with cells was transferred to the wells in row 1 on the Chromium Single Cell A Chip (10x Genomics). Single Cell 3′ Gel Beads were transferred in row 2, and Partitioning Oil was transferred into row 3. The chip was loaded on Chromium Controller to generate single-cell GEMs. GEM-RT was performed in a C1000 Touch Thermal cycler (Bio-Rad) at the following conditions: 53 °C for 45 min, 85 °C for 5 min, held at 4 °C. Post GEM-RT clean-up was performed with DynaBeads MyOne Silane Beads (ThermoFisher Scientific). cDNA was amplified using C1000 Touch Thermal cycler at the following conditions: 98 °C for 3 min; 12 cycles of 90 °C for 15 s, 67 °C for 20 s, and 72 °C for 1 min; 72 °C for 1 min, held at 4 °C. Amplified cDNA was cleaned with the SPRIselect Reagent Kit (Beckman Coulter), and quality was assessed using 2100 Bioanalyser (Agilent). Libraries were constructed following the manufacturer’s protocol and sequenced in pair-end mode on Hi-Seq4000 platform.

### Alignment and quantification of sequencing data

Cell Ranger v2.10 was used in order to de-multiplex raw base call (BCL) files generated by Illumina sequencers into FASTQ files and to perform the alignment, barcode counting, and UMI counting. Ensembl BioMart version 91 was used to generate the reference genome.

### Quality control of sequencing data

Data was filtered based on the Median Absolute Deviation (MAD) of the distribution of the number of detected genes. In addition, the percentage of mitochondrial content was set to less than 20%. Following quality control, 1764 single cells from EHT D0, 3877 from EHT D3, 2152 from EHT D5, 1742 from PD0332991 (CDK4/6i), 2140 from Roscovitine (CDK2/1i), and 1741 from RO3306 (CDK1i) were used in downstream analyses.

### Seurat alignment strategy

In order to perform a direct comparison of clusters that belonged to the same cell type across different conditions, we adopted the Seurat alignment workflow [26]. We calculated highly variable genes (HVGs) for each of the different conditions. HVGs were detected based on their average expression against their dispersion, by means of the ‘FindVariableGenes’ Seurat command with the following parameters: mean.function equal to ExpMean, dispersion.function equal to LogVMR, x.low.cutoff equal to 0.0125, x.high.cutoff equal to 3, and y.cutoff equal to 0.5. For the analysis of EHT D3 and EHT D5 we selected 1289 common HVGs that were expressed in both datasets. For the analysis of EHT D5, CDK4/6i, CDK2/1i, and CDK1i, we selected 2306 HVGs that were expressed in at least 2 out of 4 samples. Canonical correlation analysis (CCA) was then performed in order to identify shared correlation structures across the different conditions using the ‘RunMultiCCA’ command. A total of 30 and 35 CCA components were selected for the EHT D3-EHT D5 and the EHT D5-CDK4/6i-CDK2/1i-CDK1i analysis, respectively, by means of the shared correlation strength, using the ‘MetageneBicorPlot’ Seurat command. Aligned CCA space was then generated with the ‘AlignSubspace’ Seurat command. Significant CCA aligned components were then used to create the 3D tSNE space using the ‘RunTSNE’ Seurat command.

### Downstream analysis of sequencing data

For the clustering in the 3D tSNE space, we used the ‘FindClusters’ command in Seurat that performs the Shared Nearest Neighbour (SNN) modularity optimisation based clustering algorithm in correlation component analysis (CCA) aligned space. In total, thirteen clusters were identified using SNN modularity optimisation-based clustering algorithm on the significant CCA aligned components at 0.6 resolution. Positive marker genes that were expressed in at least half of the cells within the thirteen identified clusters were calculated with ‘FindAllMarkers’ Seurat command, using the Wilcoxon rank sum test with the threshold set to 0.25. We over-clustered the cells and then calculated for each cluster the average expression level of the top 20 marker genes. After calculating the correlation across the clusters, we merged those with a correlation higher than 0.9. By merging the most highly correlated, we ended up with 4 clusters, and by calculating marker genes, we were able to assign cell identity to the resulting clusters.

### Pseudotime ordering

The set of common HVGs (1289 genes) was used to order cells along a pseudotime trajectory using the Monocle2 R package v1.99.0. Monocle object was created using the ‘importCDS’ command applied to the previous step generated Seurat object. The pseudotime trajectory was generated by means of the ‘reduceDimension’ command using as additional parameters the ‘DDRTree’ reduction method, number of resulted components equal to three, and pseudo-expression equal to one, using the variance-stabilised matrix of the expression values (norm_method = ‘log’). Finally, we identified genes that change as a function of pseudotime across each of the three branches by setting the ‘fullModelFormulaStr’ parameter equal to ‘~sm.ns(Pseudotime)’. For the subclustering, we retrieved only the endothelial cluster Seurat-based defined cells (cluster 1) and performed separate Louvain clustering on the monocle space ending up with four subclusters.

### Cell cycle analysis

In order to infer cell cycle states in our single-cell data, we adopted Satija’s single-cell scoring strategy [26]. In more details, we assigned to each cell a score based on its expression of G2/M and S phase markers. These marker sets should be anticorrelated in their expression levels, and cells not expressing either are likely not cycling or in G1 phase. We assigned scores in the ‘CellCycleScoring’ function, which stores S and G2/M scores in ‘object@meta.data’, along with the predicted classification of each cell in either G2M, S, or G1 phase.

### Comparative analysis

The GEO dataset GSE135202 derived from human foetal AGM [53] was located, and the relevant raw count data from the corresponding single-cell samples GSM3993420:CS10_body_10x, GSM3993421:CS11_CH_10x, and GSM3993422:CS13_DA_10x were collected, including their annotation found in Table S3 (Cell annotation and coordinates of early and late populations), related to Figs. 1, 2, 3, and 4 of the main manuscript. In order to verify that the data were collected properly and the cell type correctly annotated and matched to the publication, we processed the raw counts using FindIntegrationAnchors and IntegrateData Seurat (v3.1) commands, as previously described [63]. Having confirmed that cells of similar cell type were clustered together forming well-defined clusters in UMAP space, we used the specific dataset as a reference and mapped the defined cell types into our data. This was performed by means of the Referenced Based Integration and Label Transfer Seurat approach [63], using FindTransferAnchors and TransferData Seurat (v3.1) commands. We set as unknown cell type cells that scored less than 30% prediction probability score. Cell types that were present in GSE135202 but not predicted in our tSNE space were also omitted from the plot. This allowed us to represent our control samples in tSNE space coloured based on GSE135202 predictions. Additionally, custom clustering analysis was performed using the Louvain clustering method (FindNeighbors and FindClusters Seurat commands) at 0.3 resolution. Finally, expression of marker genes defining key populations in Figs. 1, 2, 3, and 4 of the main manuscript were assessed against the cell clusters defined in our study.

## Supplementary information

**Additional file 1.** Supplementary Figures S1 to S10.

**Additional file 2.** Supplementary Tables S1 to S8.

**Additional file 3.** Review history.

## Data Availability

The datasets supporting the conclusions of this article are available in the ArrayExpress repository (https://www.ebi.ac.uk/arrayexpress/) under accession number E-MTAB-8205 [64]. The samples described are listed in the repository as follows: EHT D0: OT10xRNA8764427 EHT D3: EXP2_CTRL1A_4823STDY7231843 and EXP2_CTRL1B_4823STDY7231844 EHT D5: EXP2_CTRL2_4823STDY7231845 CDK4/6i: EXP2_PD033_4823STDY7231846 CDK2i: EXP2_ROSCOVITINE_4823STDY7231847 CDK1i: EXP2_RO3306_4823STDY7231848
