# Supplementary material for: Analysis of endothelial-to-haematopoietic transition at the single cell level identifies cell cycle regulation as a driver of differentiation
Source: Genome Biol. 2020 Jul 1;21:157. doi: 10.1186/s13059-020-02058-4 (PMC7329542; doi:10.1186/s13059-020-02058-4)
Supplement: Supplementary file 1 — Additional file 1. Supplementary Figures S1 to S10. [file 13059_2020_2058_MOESM1_ESM.pdf]

Fig. S1

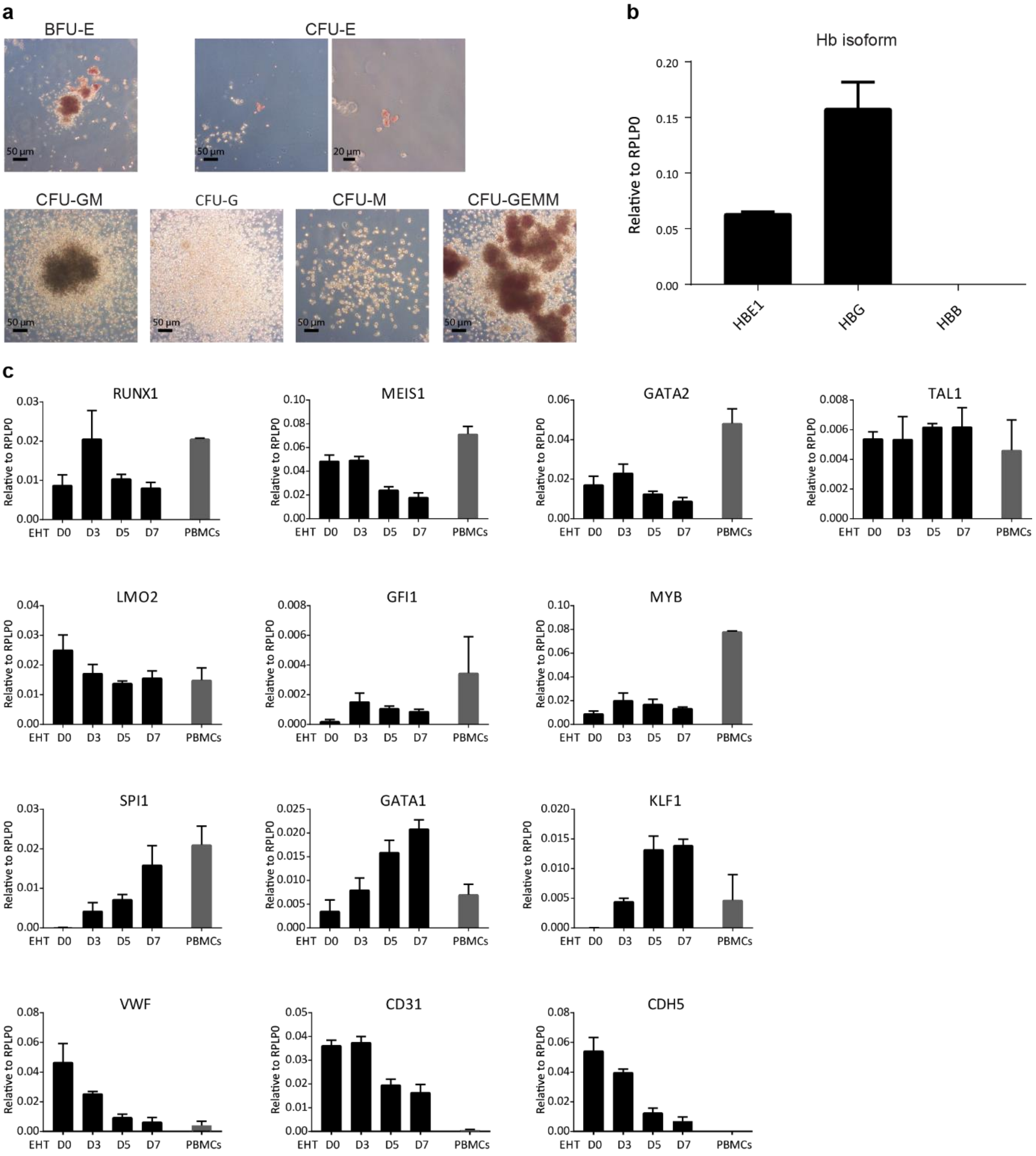

Fig. S1 Characterisation of the differentiation system. (A) Colonies generated by CFU assay from cells collected at EHT D5. Scale bar as indicated. BFU-E: burst forming unit-erythroid, CFU-E: colony forming unit-erythroid, CFU-GM: colony forming unit-granulocyte-macrophage, CFU-G: colony forming unit-granulocyte, CFU-M: colony forming unit-macrophage, CFU-GEMM: colony forming unit-granulocyte-erythrocyte-macrophage-megakaryocyte. **(B)** qPCR analysis of cells collected after CFU assay, showing the expression of different haemoglobin isoform genes. Data are means ±SEM of 3 independent experiments. **(C)** qPCR analysis of cells collected at different time points during EHT, compared to primary CD34+ cells isolated from peripheral blood. PBMC: peripheral blood mononuclear cells. Data are means ±SEM of 3 independent experiments for EHT samples and 2 independent samples for PBMCs.

Fig. S2

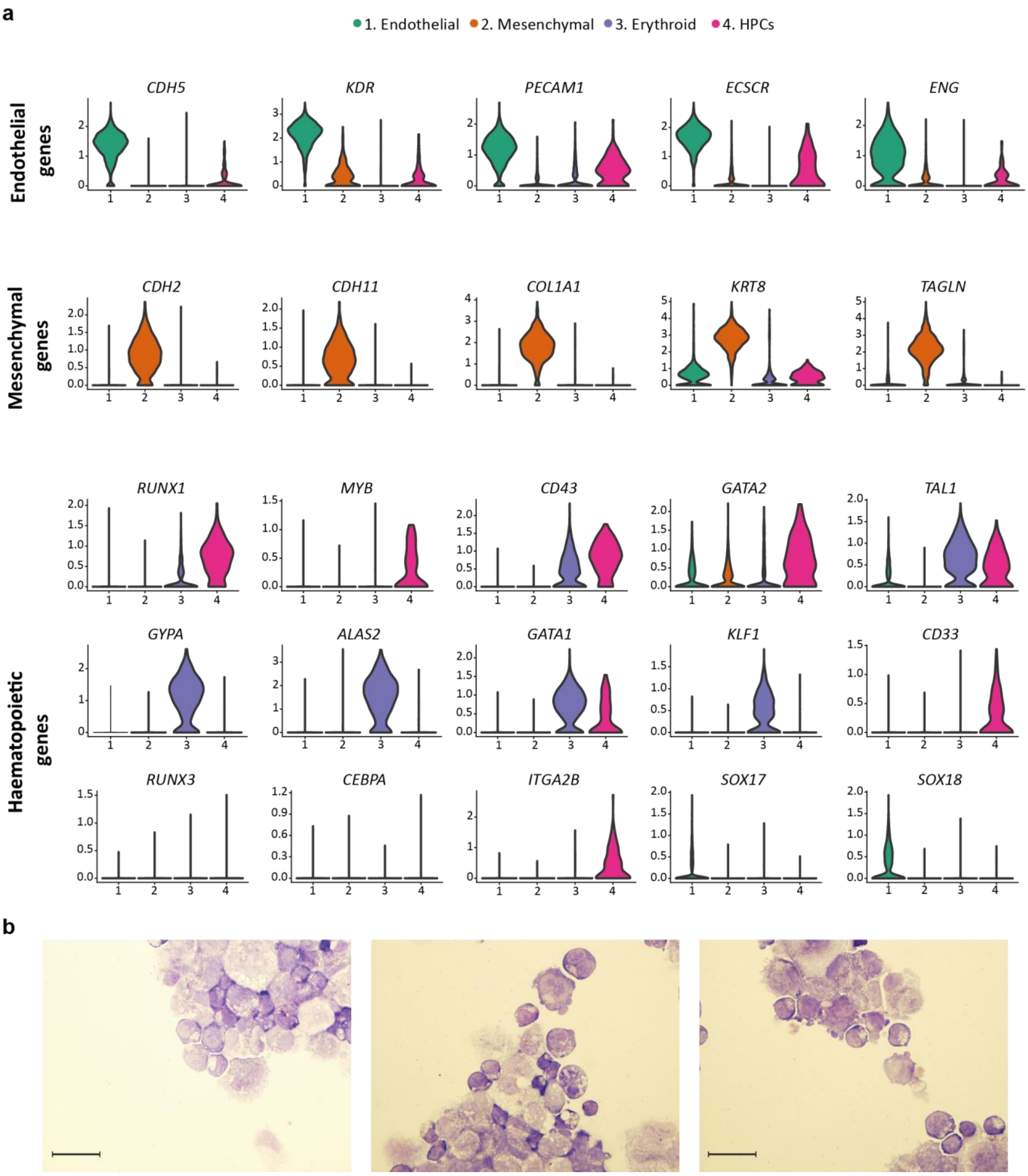

Fig. S2 Expression of lineage marker genes in the scRNA-seq dataset. (A) Violin plots showing the expression of key lineage markers per cluster. The analysis refers to merged data from EHT D3 and EHT D5 samples. Plots are log2 scaled of the counts. **(B)** May-Grünwald-Giemsa staining of cells collected at EHT D5 and prepared on microscopy slides by cytospin centrifugation. The assay revealed immature cell populations with the absence of obvious morphologies indicating terminally differentiated blood cells in culture, consistent with previous studies of mouse haemogenic endothelium during EHT from E11.5 AGM [Taoudi *et al.*, 2005]. Scale bar is 15 µm.

Fig. S3

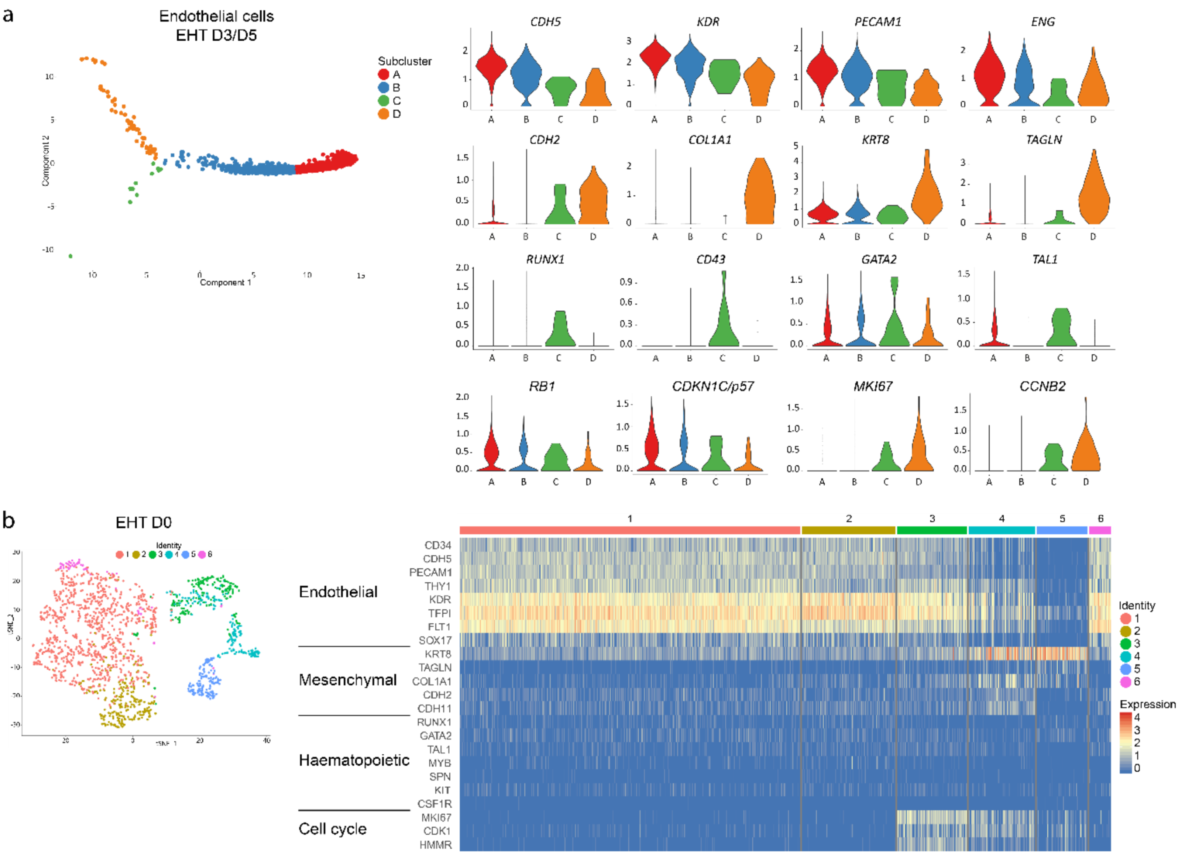

Fig. S3 Dissection of endothelial cell commitment. (A) Subclustering of the endothelial population. (Left panel) Merged data from EHT D3 and EHT D5 showing differentiation trajectory for endothelial cells only. Cells were coloured to indicate distinct subclusters. (Right panel) Violin plots showing the expression of key lineage and cell cycle genes on endothelial subclusters. Plots are log2 scaled of the counts. All the cells composing the trajectory in this figure are endothelial cells from the endothelial cell cluster (Fig. 2), and violin plots show the expression of different genes in different subsets of the endothelial cluster. **(B)** scRNA-seq analysis of cells sorted at EHT D0. (Left panel) tSNE plot showing cells sorted as CD34+/CD43- and used for the subsequent EHT culture. Six populations were identified based on the transcriptional profiles. (Right panel) Heatmap showing the expression of lineage-associated and cell cycle genes on each cluster. The majority of the cells analysed express genes consistent with an endothelial identity; haematopoietic genes are not expressed at this stage; a small fraction of cells (cluster 4, and to a minor extent cluster 3) co-express endothelial and mesenchymal genes. This also corresponds to the expression of cell cycle related genes.

Fig. S4

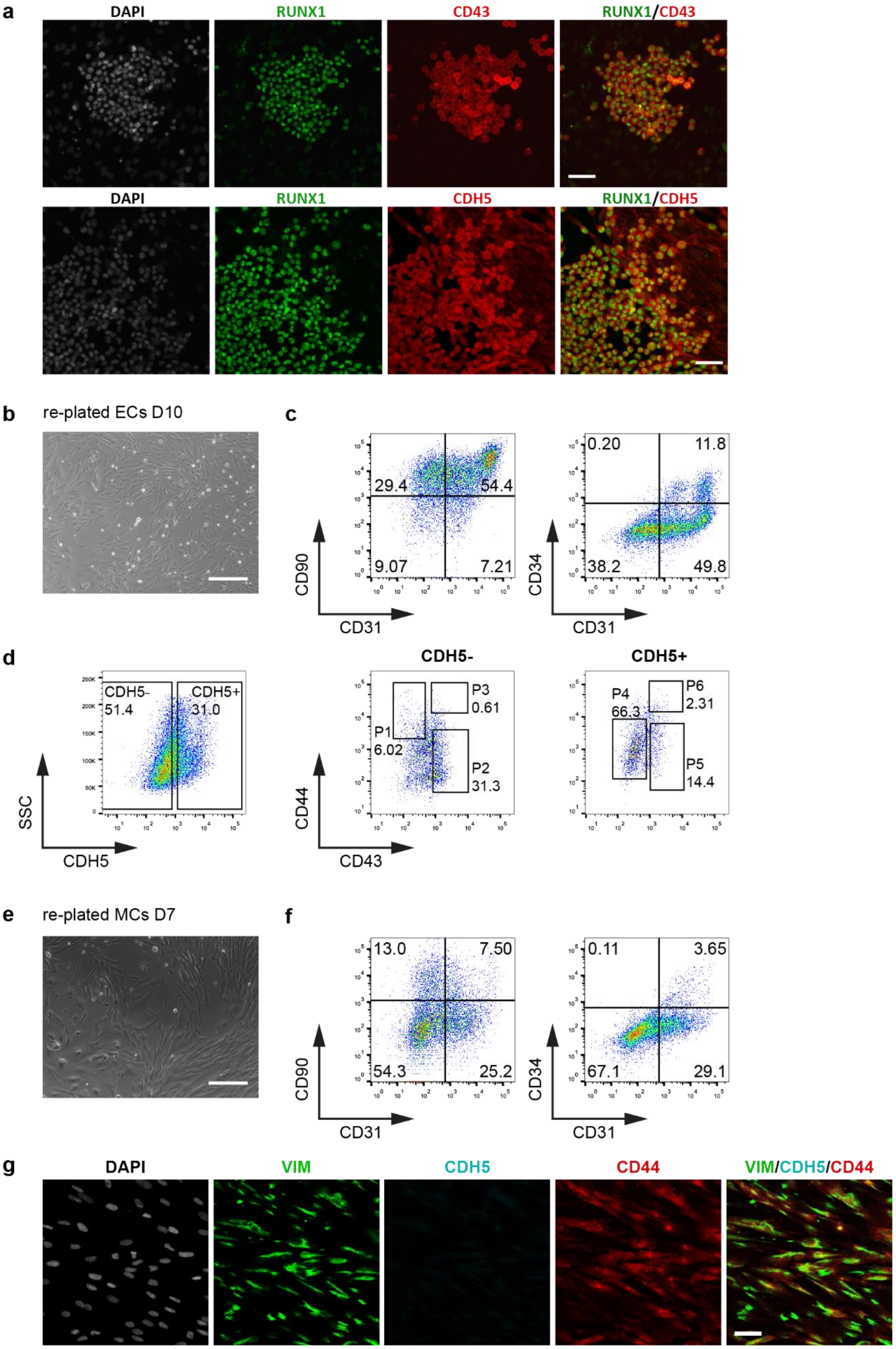

Fig. S4 Characterisation and validation of distinct populations. (A) Immunofluorescence images of samples at EHT D5, showing co-expression of RUNX1 and CD43 (upper panel) or

RUNX1 and CDH5 (lower panel) in haematopoietic clusters. Cells showing dim RUNX1 expression but CD43 negative (upper panel) or CDH5 positive (lower panel) possibly represent haemogenic endothelial cells prior to the haematopoietic transition. Scale bar is 50 μm. **(B)** Representative image of endothelial cells (ECs) sorted at EHT D3 as CDH5+/CD44+/CD43- (P4 in Fig. 3) and grown for 10 days. Scale bar is 200 μm. **(C)** The majority of these cells are positive for the endothelial markers CD31 and CD90, with some of the cells positive for CD34. **(D)** Importantly, these re-plated culture also contains HPCs (P3 and P6), erythroid cells (P2 and P5) and mesenchymal cells (P1), confirming that sorted endothelial cells are able to generate the described populations. **(E)** Representative image of mesenchymal cells (MCs) sorted at EHT D3 as CDH5-/CD44high/CD43- (P1 in Fig. 3) and grown for 7 days. Scale bar is 200 μm. **(F)** Most of these cells appear negative or low positive for endothelial markers CD31 and CD90, with very few double positive events. **(G)** Immunofluorescence staining demonstrates that these cells are also negative for CDH5, but positive for CD44 and Vimentin (VIM), confirming their mesenchymal identity. Results in B-G are representative of 2 independent experiments.

Fig. S5

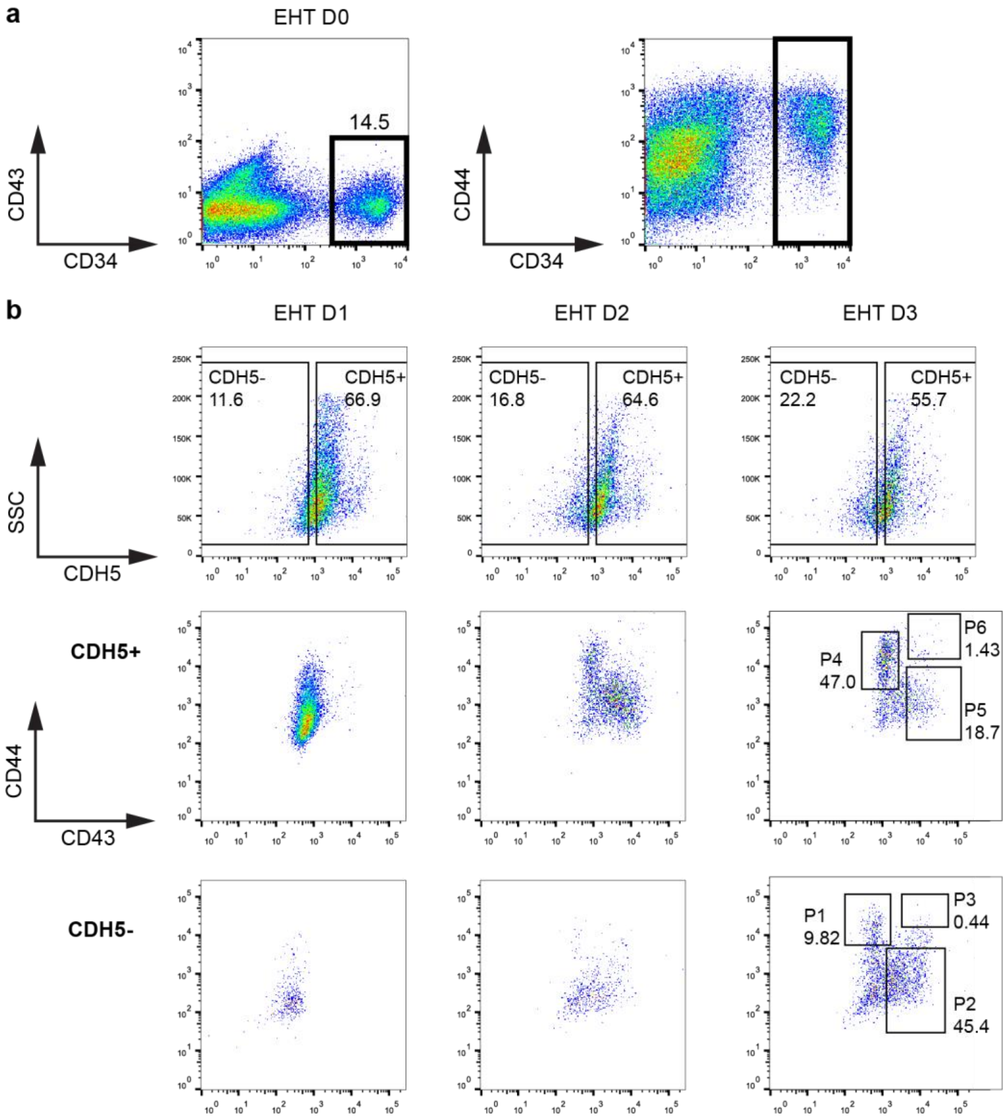

Fig. S5 Time course during EHT. (A) Cells sorted at EHT D0 as CD34+/CD43- (left) were also shown to be CD44low/+ (right). Black box shows corresponding populations in the two panels. **(B)** The sorted population was followed through EHT to monitor the expression of CDH5, CD43 and CD44. This showed that a starting population of CDH5+/CD44low/+ cells gradually acquires brighter expression of CD44 and generate on one side CD43+ cells (HPCs and erythroid), on the other CD44high/CDH5- cells (mesenchymal). Results are representative of 2 independent experiments.

Fig S6

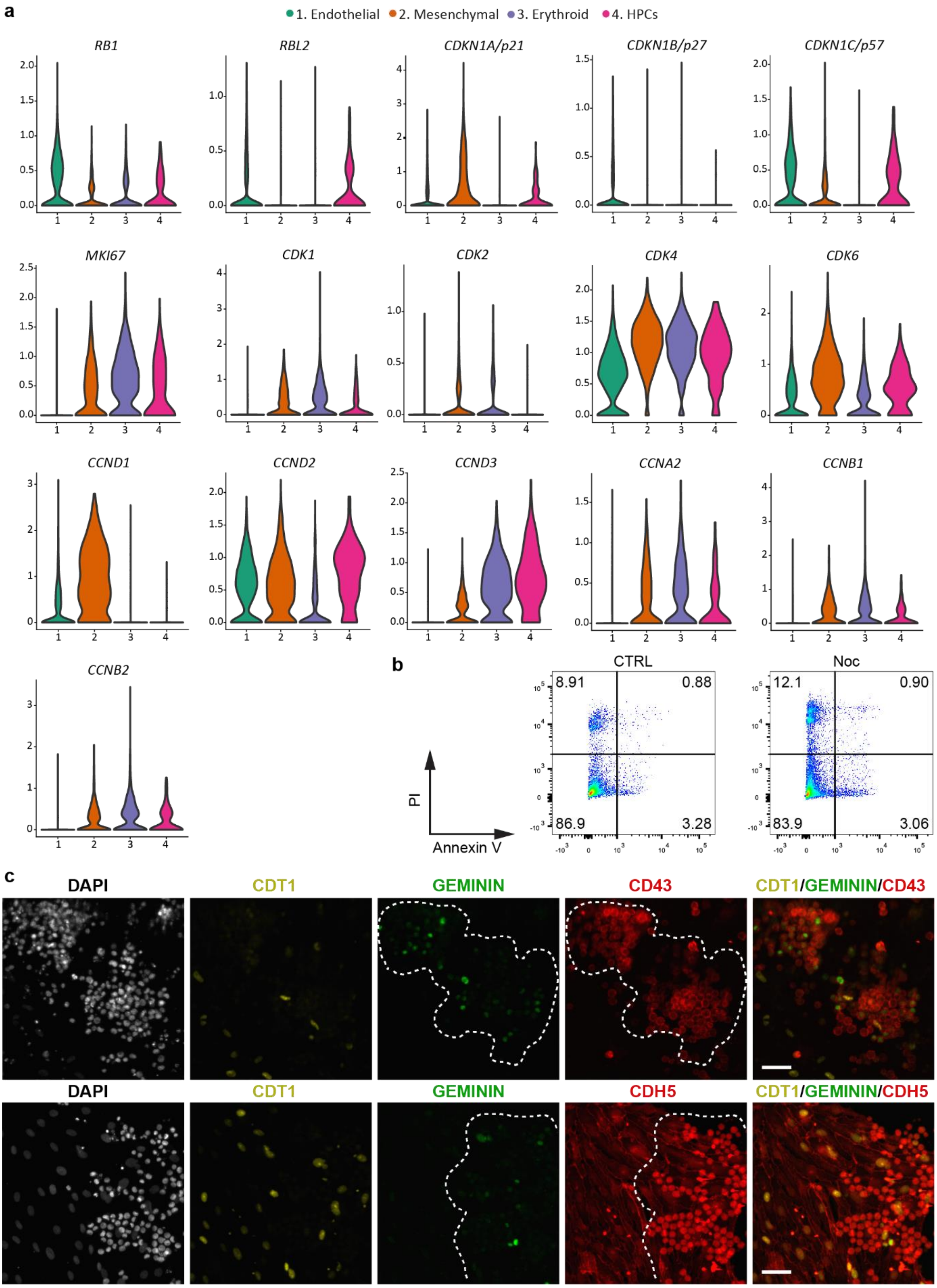

Fig. S6 EHT is associated with cell cycle entry. (A) Violin plots showing the expression of key cell cycle regulators in distinct clusters (as defined in Fig. 2). Merged data from EHT D3 and EHT D5, plots are log2 scaled of the counts. **(B)** Apoptosis and cell death staining of

Nocodazole treated cultures. At EHT D3, media was supplemented either with 0.1% DMSO (CTRL) or with 0.1 µg/mL Nocodazole (Noc). After 48h, at EHT D5 cells were washed to remove Nocodazole as in previous experiments. Cells were collected after further 48h in culture to detect any long-term toxic effect of the treatments and stained for Annexin V and Propidium Iodide (PI) to monitor apoptotic (Annexin V+) and dead cells (PI+). **(C)** Immunofluorescence images of differentiated FUCCI-hPSCs at EHT D5. The Geminin reporter is expressed during S/G2/M phase and is only expressed in haematopoietic clusters (CD43 positive in upper panel, CDH5 positive and round shaped in lower panel). The CDT1 reporter is expressed during late G1 and is mainly expressed in cells surrounding haematopoietic clusters. Cells in G0/early G1 are negative for both reporters. Scale bar is 50 µm.

Fig. S7

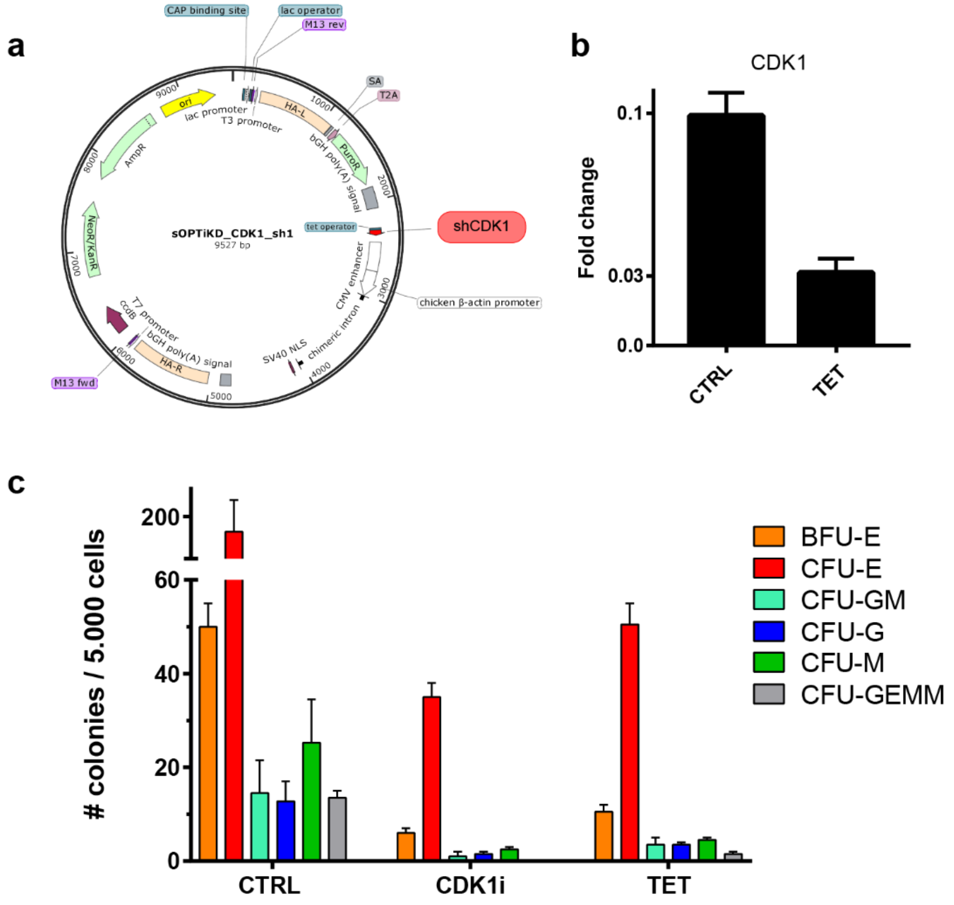

Fig. S7 Validation of the role of CDK1 by inducible knockdown. (A) Genetic tool that allows the inducible expression of a shRNA upon addition of tetracyclin (TET) in culture for the inducible knockdown of CDK1. **(B)** Cells were treated with TET for 3 days starting at EHT D2. qPCR analysis shows that the induction leads to a 70% reduction of CDK1 mRNA. **(C)** Cells were either supplemented at EHT D3 with 10 µM RO3306 (CDK1i) for 48h, or at EHT D2 with 0.1% DMSO (CTRL) or 1 µg/ml tetracycline for 72h. At EHT D5, cells were collected, washed and cultured in a CFU assay to assess their differentiation potential. Results are representative of 2 independent experiments.

Fig. S8

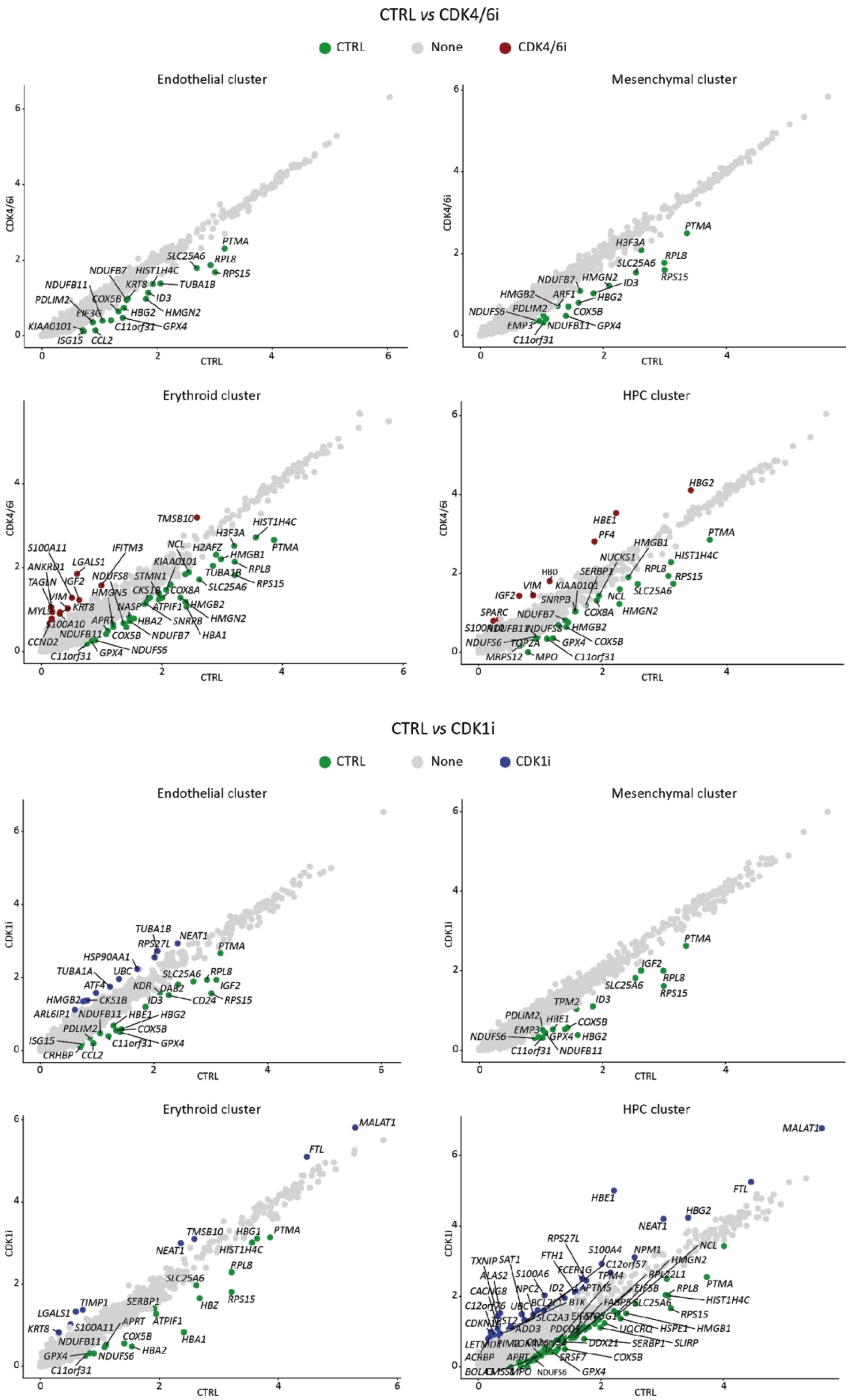
CTRL vs CDK4/6i
CTRL
None
CDK4/6i
Endothelial cluster
Mesenchymal cluster
Erythroid cluster
HPC cluster
CDK4/6i
CTRL
PTMA
SLC25A6
RPL8
RPS15
HIST1H4C
TUBA1B
NDUFB7
NDUFB11
KRT8
ID3
PDLIM2
COX5B
HBG2
HMGN2
KIAA0101
C11orf31
GPX4
ISG15
CCL2
H3F3A
NDUFS6
EMP3
HMGB2
TMSB10
HBE1
PF4
HMGB1
NCL
MPO
CTRL vs CDK1i
CDK1i
TUBA1B
NEAT1
RPS27L
HSP90AA1
MALAT1
FTL
HBG1
NPM1
S100A4
TXNIP
SAT1
HIST1H4C
CD24
IGF2
DAB2
KDR
LGALS1
TIMP1
SERBP1
APRT
ATPIF1
HBA1
HBA2
HBZ

Fig. S8 Effect of CDK inhibitors on gene expression in each cluster. Regression plots showing differentially expressed genes per cluster between treatment and control. For each gene and on each sample, axes are log2 of the average counts. Differentially expressed genes are highlighted. Cut-off used is log2 fold change = 0.5.

Fig. S9

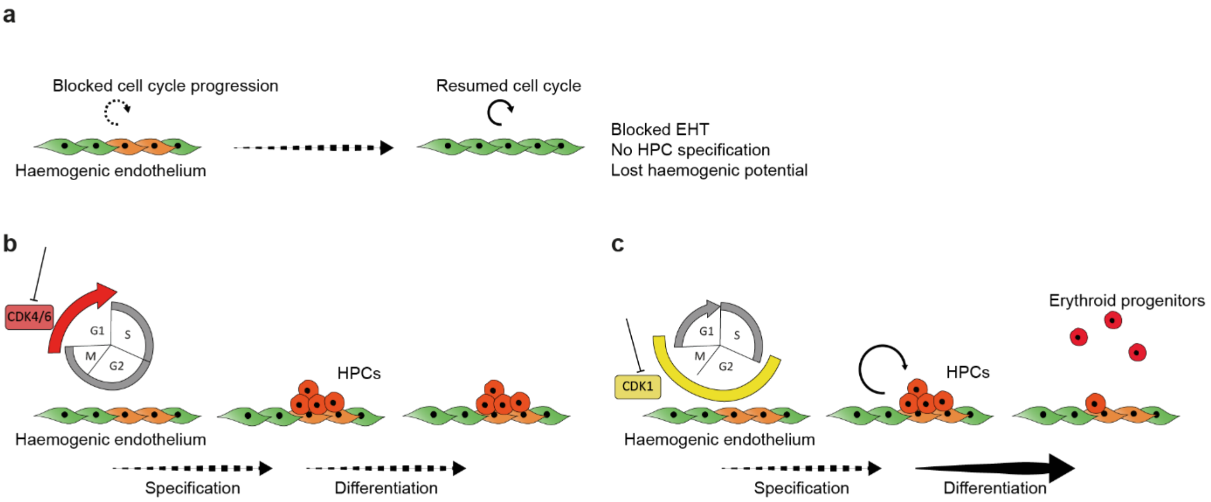

Fig. S9 Proposed model for the role of cell cycle during EHT. (A) Cell cycle progression is needed for endothelial cells to progress to the haematopoietic fate. If cell cycle is shortly blocked and subsequently released, the haemogenic potential is lost and endothelial cells start proliferating as a monolayer. **(B)** CDK4/6 is important for EHT and for HPC ability to further differentiate. **(C)** CDK1 is important for EHT and for HPC maintenance and survival.

Fig. S10

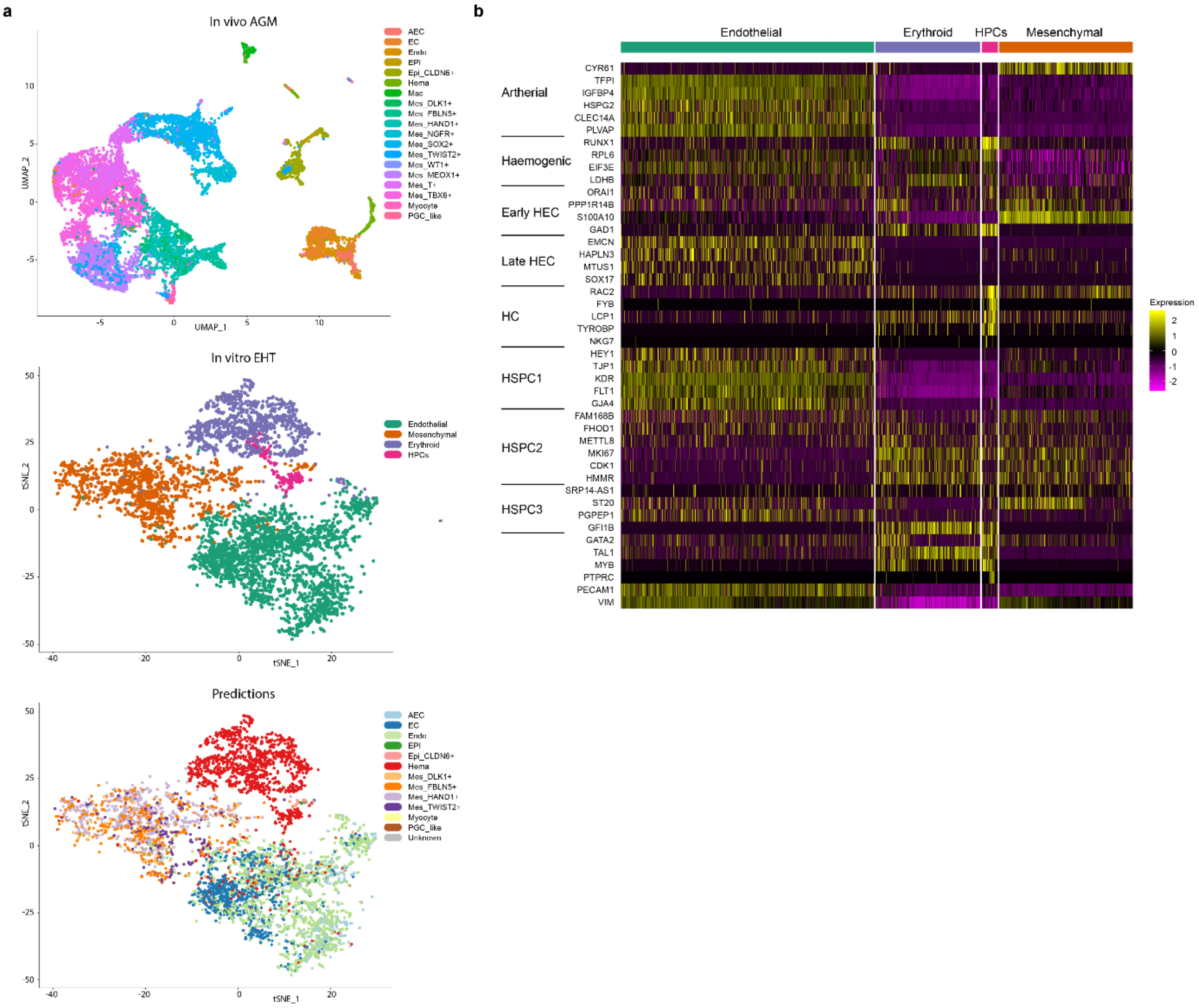

Fig. S10 scRNA-seq reveals similarities between *in vitro* EHT culture and *in vivo* AGM. (A) Dataset from human AGM (**64**) was annotated according to Authors' original annotations. These were then used to predict cell identities in our scRNA-seq dataset to detect similarities between *in vitro* and *in vivo* generated cell types. The analysis confirmed that endothelial, mesenchymal and haematopoietic cells identified in our dataset displayed similarity with corresponding populations described in vivo **(B)** Heatmap of the marker genes defining different populations *in vivo*, showing their expression in the clusters originally identified in our dataset. Early/Late HEC: early/late haemogenic endothelial cells, HC: haematopoietic cells, HSPC1-3: haematopoietic stem/progenitor cells type 1-3. Similarly to AGM-derived haemogenic endothelial cells, endothelial cells generated *in vitro* expressed both haemogenic and arterial genes. Additionally, *in vitro* derived endothelial cells displayed higher similarity with late HECs, from which true HSPCs are generated in the AGM, rather than to early HECs. HPCs generated *in vitro* displayed similarities with immature HSPC2 and end stage HSPC3 populations.
