# Supplementary material for: Analysis of endothelial-to-haematopoietic transition at the single cell level identifies cell cycle regulation as a driver of differentiation
Source: Genome Biol. 2020 Jul 1;21:157. doi: 10.1186/s13059-020-02058-4 (PMC7329542; doi:10.1186/s13059-020-02058-4)
Supplement: Supplementary file 2 — Additional file 2. Supplementary Tables S1 to S8. [file 13059_2020_2058_MOESM2_ESM.pdf]

Table S1 GO terms for CDK4/6i differentially expressed genes in the endothelial cluster.

**Endothelial cluster | CDK4/6i downregulated genes | GO Biological Process**				
Name	p-value	adjusted p-value	z score	combined score
ribosomal small subunit export from nucleus (GO:0000056)	0.00698	0.05914	-4.17	20.7
mitochondrial ATP synthesis coupled proton transport (GO:0042776)	0.02178	0.07542	-4.96	18.99
mitochondrial electron transport, NADH to ubiquinone (GO:0006120)	0.001	0.04224	-2.73	18.88
mitochondrial ATP synthesis coupled electron transport (GO:0042775)	8.3E-05	0.006288	-1.9	17.87
viral process (GO:0016032)	6.1E-05	0.006288	-1.7	16.45
response to type I interferon (GO:0034340)	0.00797	0.05914	-2.97	14.36
positive regulation of apoptotic cell clearance (GO:2000427)	0.00797	0.05914	-2.78	13.42
ribosomal subunit export from nucleus (GO:0000054)	0.01293	0.06483	-3.08	13.38
regulation of glial cell apoptotic process (GO:0034350)	0.00698	0.05914	-2.65	13.17
positive regulation of nitric-oxide synthase biosynthetic process (GO:0051770)	0.00996	0.06483	-2.79	12.84

Table S2 GO terms for CDK4/6i differentially expressed genes in the HPC cluster.

**HPC cluster | CDK4/6i downregulated genes | GO Biological Process**				
Name	p-value	adjusted p-value	z score	combined score
mitochondrial electron transport, NADH to ubiquinone (GO:0006120)	2.75E-07	0.00001807	-2.73	41.3
mitochondrial ATP synthesis coupled electron transport (GO:0042775)	6.70E-10	1.62E-07	-1.9	40.19
DNA topological change (GO:0006265)	1.81E-07	0.00001588	-2.48	38.54
V(D)J recombination (GO:0033151)	0.00003847	0.0008432	-3.28	33.34
respiratory electron transport chain (GO:0022904)	1.23E-09	1.62E-07	-1.36	27.89
DNA ligation (GO:0006266)	0.000001453	0.00004245	-2.07	27.79
apoptotic nuclear changes (GO:0030262)	0.000003022	0.00007949	-1.91	24.29
DNA ligation involved in DNA repair (GO:0051103)	0.0001068	0.00216	-2.5	22.89
mitochondrial respiratory chain complex I biogenesis (GO:0097031)	0.000001028	0.00003862	-1.58	21.76
NADH dehydrogenase complex assembly (GO:0010257)	0.000001028	0.00003862	-1.57	21.7

**HPC cluster | CDK4/6i upregulated genes | GO Biological Process**				
Name	p-value	adjusted p-value	z score	combined score
regulated exocytosis (GO:0045055)	0.00002208	0.001921	-2.3	24.7
membrane raft assembly (GO:0001765)	0.002797	0.03577	-3.89	22.88
defense response to protozoan (GO:0042832)	0.003595	0.03577	-2.98	16.76
platelet degranulation (GO:0002576)	0.00001305	0.001921	-1.44	16.15
membrane raft organization (GO:0031579)	0.004392	0.03577	-2.96	16.05
regulation of MHC class II biosynthetic process (GO:0045346)	0.003595	0.03577	-2.73	15.38
positive regulation of cAMP-mediated signaling (GO:0043950)	0.003595	0.03577	-2.7	15.2
positive regulation of macrophage differentiation (GO:0045651)	0.003595	0.03577	-2.59	14.56
genetic imprinting (GO:0071514)	0.003595	0.03577	-2.57	14.45
regulation of glycogen (starch) synthase activity (GO:2000465)	0.003196	0.03577	-2.44	14.04

Table S3 GO terms for CDK4/6i differentially expressed genes in the erythroid cluster.

**Erythroid cluster | CDK4/6i downregulated genes | GO Biological Process**				
Name	p-value	adjusted p-value	z score	combined score
mitochondrial ATP synthesis coupled electron transport (GO:0042775)	2.92E-10	1.25E-07	-1.9	41.77
mitochondrial electron transport, NADH to ubiquinone (GO:0006120)	0.000002073	0.0002228	-2.73	35.77
V(D)J recombination (GO:0033151)	0.000103	0.00492	-3.28	30.13
respiratory electron transport chain (GO:0022904)	5.92E-10	1.27E-07	-1.36	28.88
mitochondrial respiratory chain complex assembly (GO:0033108)	0.000001286	0.0001843	-1.78	24.16
DNA topological change (GO:0006265)	0.0001651	0.007099	-2.47	21.54
DNA geometric change (GO:0032392)	0.00001531	0.0008227	-1.87	20.72
DNA ligation involved in DNA repair (GO:0051103)	0.0002851	0.009431	-2.5	20.44
mitochondrial respiratory chain complex I biogenesis (GO:0097031)	0.000007671	0.0004712	-1.58	18.59
NADH dehydrogenase complex assembly (GO:0010257)	0.000007671	0.0004712	-1.57	18.54

**Erythroid cluster | CDK4/6i upregulated genes | GO Biological Process**				
Name	p-value	adjusted p-value	z score	combined score
membrane raft assembly (GO:0001765)	0.004193	0.05535	-3.89	21.31
regulation of viral entry into host cell (GO:0046596)	0.0001149	0.02343	-1.89	17.14
regulation of muscle system process (GO:0090257)	0.005985	0.05535	-2.94	15.05
membrane raft organization (GO:0031579)	0.006582	0.05535	-2.96	14.86
genetic imprinting (GO:0071514)	0.005388	0.05535	-2.57	13.41
response to muscle stretch (GO:0035994)	0.005985	0.05535	-2.57	13.14
regulation of glycogen (starch) synthase activity (GO:2000465)	0.004791	0.05535	-2.44	13.05
negative regulation of DNA metabolic process (GO:0051053)	0.0003079	0.0314	-1.52	12.28
regulation of apoptotic process (GO:0042981)	0.001047	0.05535	-1.79	12.27
positive regulation of G1/S transition of mitotic cell cycle (GO:1900087)	0.01016	0.05535	-2.66	12.22

Table S4 GO terms for CDK4/6i differentially expressed genes in the mesenchymal cluster.

**Mesenchymal cluster | CDK4/6i downregulated genes | GO Biological Process**				
Name	p-value	adjusted p-value	z score	combined score
mitochondrial electron transport, NADH to ubiquinone (GO:0006120)	0.000009682	0.000568	-2.74	31.58
mitochondrial ATP synthesis coupled electron transport (GO:0042775)	9.31E-07	0.0001222	-1.9	26.42
ribosomal small subunit export from nucleus (GO:0000056)	0.006284	0.04096	-4.16	21.09
mitochondrial ATP synthesis coupled proton transport (GO:0042776)	0.01962	0.05571	-4.94	19.43
respiratory electron transport chain (GO:0022904)	0.000001389	0.0001222	-1.36	18.33
mitochondrial respiratory chain complex I biogenesis (GO:0097031)	0.00002582	0.0007574	-1.58	16.68
NADH dehydrogenase complex assembly (GO:0010257)	0.00002582	0.0007574	-1.57	16.64
mitochondrial respiratory chain complex assembly (GO:0033108)	0.00008825	0.002219	-1.78	16.58
V(D)J recombination (GO:0033151)	0.007179	0.04211	-3.26	16.12
mitochondrial respiratory chain complex I assembly (GO:0032981)	0.00002582	0.0007574	-1.41	14.9

Table S5 GO terms for CDK1i differentially expressed genes in the endothelial cluster.

**Endothelial cluster | CDK1i downregulated genes | GO Biological Process**				
Name	p-value	adjusted p-value	z score	combined score
positive regulation of nitric-oxide synthase biosynthetic process (GO:0051770)	0.0000383	0.01255	-2.82	28.65
regulation of nitric-oxide synthase biosynthetic process (GO:0051769)	0.00006628	0.01255	-2.58	24.83
ribosomal small subunit export from nucleus (GO:0000056)	0.006632	0.05774	-4.15	20.81
mitochondrial ATP synthesis coupled proton transport (GO:0042776)	0.0207	0.05998	-4.86	18.84
positive regulation of activated T cell proliferation (GO:0042104)	0.0001608	0.01267	-2.15	18.76
positive regulation of cell migration by vascular endothelial growth factor signaling pathway (GO:0038089)	0.007576	0.05774	-3.47	16.95
regulation of positive chemotaxis (GO:0050926)	0.008519	0.05774	-3.55	16.93
protein kinase B signaling (GO:0043491)	0.0005284	0.02313	-1.97	14.87
response to type I interferon (GO:0034340)	0.007576	0.05774	-2.95	14.4
positive regulation of apoptotic cell clearance (GO:2000427)	0.007576	0.05774	-2.76	13.47

**Endothelial cluster | CDK1i upregulated genes | GO Biological Process**				
Name	p-value	adjusted p-value	z score	combined score
cytoskeleton-dependent intracellular transport (GO:0030705)	0.00007845	0.006058	-2.09	19.73
regulation of transcription from RNA polymerase II promoter in response to oxidative stress (GO:0043619)	0.004492	0.04395	-3.6	19.45
cell cycle G2/M phase transition (GO:0044839)	0.00002973	0.005055	-1.82	18.98
V(D)J recombination (GO:0033151)	0.003994	0.04395	-3.27	18.05
regulation of mitotic cell cycle phase transition (GO:1901990)	0.00008908	0.006058	-1.91	17.85
outer mitochondrial membrane organization (GO:0007008)	0.003994	0.04395	-3.23	17.84
regulation of G2/M transition of mitotic cell cycle (GO:0010389)	0.00004774	0.005411	-1.77	17.61
negative regulation of translation in response to stress (GO:0032055)	0.003495	0.04395	-3.09	17.48
regulation of translational initiation in response to stress (GO:0043558)	0.003495	0.04395	-2.84	16.07
regulation of nuclease activity (GO:0032069)	0.003495	0.04395	-2.83	15.99

Table S6 GO terms for CDK1i differentially expressed genes in the HPC cluster.

HPC cluster | CDK1i downregulated genes | GO Biological Process				
Name	p-value	adjusted p-value	z score	combined score
mitochondrial ATP synthesis coupled electron transport (GO:0042775)	8.30E-11	4.67E-08	-1.9	44.16
mitochondrial electron transport, NADH to ubiquinone (GO:0006120)	0.000001458	0.0001368	-2.73	36.68
respiratory electron transport chain (GO:0022904)	2.06E-10	5.79E-08	-1.36	30.32
mitochondrial ATP synthesis coupled proton transport (GO:0042776)	0.003724	0.03913	-4.95	27.71
mitochondrial electron transport, cytochrome c to oxygen (GO:0006123)	0.0001003	0.002822	-2.57	23.62
mRNA splicing, via spliceosome (GO:0000398)	0.000001398	0.0001368	-1.75	23.54
histone mRNA metabolic process (GO:0008334)	0.0001484	0.003632	-2.29	20.16
gene expression (GO:0010467)	1.29E-07	0.00002419	-1.26	19.94
establishment of protein localization to mitochondrion (GO:0072655)	0.00005491	0.001932	-2.02	19.86
nucleocytoplasmic transport (GO:0006913)	0.00004293	0.001859	-1.95	19.59
**HPC cluster | CDK1i upregulated genes | GO Biological Process**				
Name	p-value	adjusted p-value	z score	combined score
cellular iron ion homeostasis (GO:0006879)	0.000003262	0.0008498	-2.65	33.52
iron ion homeostasis (GO:0055072)	9.99E-08	0.00005206	-1.57	25.36
oxygen homeostasis (GO:0032364)	0.01253	0.09524	-4.65	20.36
positive regulation of cell cycle arrest (GO:0071158)	0.00001468	0.001912	-1.71	19.01
ribosomal small subunit export from nucleus (GO:0000056)	0.01253	0.09524	-4.12	18.06
gas homeostasis (GO:0033483)	0.01431	0.09524	-4.11	17.47
neutrophil degranulation (GO:0043312)	0.0001957	0.01014	-2.01	17.14
cellular transition metal ion homeostasis (GO:0046916)	0.00002402	0.002503	-1.59	16.88
neutrophil mediated immunity (GO:0002446)	0.000214	0.01014	-1.93	16.31
DNA damage response, signal transduction by p53 class mediator (GO:0030330)	0.00001468	0.001912	-1.42	15.84

Table S7 GO terms for CDK1i differentially expressed genes in the erythroid cluster.

**Erythroid cluster | CDK1i downregulated genes | GO Biological Process**				
Name	p-value	adjusted p-value	z score	combined score
regulation of protein targeting (GO:1903533)	0.005936	0.03332	-4.26	21.86
ribosomal small subunit export from nucleus (GO:0000056)	0.005936	0.03332	-4.17	21.37
mitochondrial electron transport, NADH to ubiquinone (GO:0006120)	0.0007188	0.02581	-2.74	19.8
mitochondrial ATP synthesis coupled proton transport (GO:0042776)	0.01854	0.04191	-4.96	19.77
mitochondrial ATP synthesis coupled electron transport (GO:0042775)	0.00004997	0.003801	-1.9	18.84
folic acid-containing compound biosynthetic process (GO:0009396)	0.005936	0.03332	-3.45	17.69
oxygen transport (GO:0015671)	0.006781	0.03332	-3.32	16.57
positive regulation of autophagy of mitochondrion (GO:1903599)	0.006781	0.03332	-3	15
ribosomal subunit export from nucleus (GO:0000054)	0.011	0.04056	-3.08	13.89
pteridine-containing compound biosynthetic process (GO:0042559)	0.006781	0.03332	-2.68	13.41

**Erythroid cluster | CDK1i upregulated genes | GO Biological Process**				
Name	p-value	adjusted p-value	z score	combined score
negative regulation of membrane protein ectodomain proteolysis (GO:0051045)	0.003196	0.04232	-2.66	15.26
negative regulation of reproductive process (GO:2000242)	0.004392	0.04232	-2.54	13.81
sequestering of actin monomers (GO:0042989)	0.004392	0.04232	-2.43	13.17
regulation of integrin-mediated signaling pathway (GO:2001044)	0.004791	0.04232	-2.3	12.27
regulation of trophoblast cell migration (GO:1901163)	0.004791	0.04232	-2.24	11.96
negative regulation of DNA replication (GO:0008156)	0.006781	0.04303	-2.2	10.96
positive regulation of viral entry into host cell (GO:0046598)	0.003595	0.04232	-1.85	10.44
transition metal ion homeostasis (GO:0055076)	0.009958	0.04303	-2.23	10.26
cellular iron ion homeostasis (GO:0006879)	0.02258	0.0497	-2.61	9.91
negative regulation of actin filament polymerization (GO:0030837)	0.009165	0.04303	-1.83	8.57

Table S8 GO terms for CDK1i differentially expressed genes in the mesenchymal cluster.

**Mesenchymal cluster | CDK1i downregulated genes | GO Biological Process**				
Name	p-value	adjusted p-value	z score	combined score
ribosomal small subunit export from nucleus (GO:0000056)	0.005587	0.0459	-4.17	21.63
mitochondrial electron transport, NADH to ubiquinone (GO:0006120)	0.0006352	0.0305	-2.74	20.14
mitochondrial ATP synthesis coupled proton transport (GO:0042776)	0.01746	0.05732	-4.96	20.08
mitochondrial ATP synthesis coupled electron transport (GO:0042775)	0.00004128	0.004197	-1.9	19.21
bleb assembly (GO:0032060)	0.006383	0.0459	-3	15.15
ribosomal subunit export from nucleus (GO:0000054)	0.01035	0.05579	-3.08	14.08
respiratory electron transport chain (GO:0022904)	0.00005559	0.004197	-1.36	13.32
genetic imprinting (GO:0071514)	0.007178	0.04927	-2.56	12.64
regulation of glycogen (starch) synthase activity (GO:2000465)	0.006383	0.0459	-2.43	12.27
protein targeting to ER (GO:0045047)	0.002727	0.03956	-2.08	12.26
