# Supplementary material for: Analysis of endothelial-to-haematopoietic transition at the single cell level identifies cell cycle regulation as a driver of differentiation
Source: Genome Biol. 2020 Jul 1;21:157. doi: 10.1186/s13059-020-02058-4 (PMC7329542; doi:10.1186/s13059-020-02058-4)
Supplement: Supplementary file 3 — Additional file 3. Review history. [file 13059_2020_2058_MOESM3_ESM.docx]

Review History

**First round of review**

**Reviewer 1**

**Are you able to assess all statistics in the manuscript, including the appropriateness of statistical tests used?** No, I do not feel adequately qualified to assess the statistics.

**Comments to author:**

In this study Dr Cvejic and colleagues investigated the role of cell proliferation in the endothelial-to-hemopoietic transition (EHT) in human ES cells. They found that inhibiting proliferation supresses the emergence of blood cells and concluded that it is essential for EHT. The paper contains a lot of data but is difficult to follow due to deviations and often inaccurate statements. My main concerns are:

(1)     The authors carried out a single cell transcriptomics analysis to infer differentiation trajectory of ES cells using Monocle software. The trajectory has 3 branches of mesodermal, endothelial and hemopoietic cells. They claim that this shows that endothelial cells generate both mesodermal and hemopoietic cells whereas in fact Monocle does not determine directions. This plot is completely compatible with the more standard view of mesodermal cells transitioning into endothelial and subsequently blood cells. The authors should validate cell fates in these 3 branches by culturing sorted populations in appropriate conditions (not just hemopoietic methylcellulose assay). I am not sure that Monocle graph and PCA analysis add any useful information to our understanding of EHT compared to what was known before. Overall this part contains a lot of details which are disentangled from the main analysis of the role of cell cycle in EHT. For example, it is not clear how pictures of cell clusters under the microscope or speculations about localisation of CDH5+ and CD5- cells in culture help understand the role of proliferation in EHT.

(2)     To determine effects of cell proliferation on EHT they treated entire cultures (which contain several types of cells including hemopoietic progenitors, HPC) with various cell cycle chemical inhibitors. They observed fewer blood cells but more endothelial cells and concluded that proliferation is important for EHT. However, this might be due to inhibition of HPC expansion which already present in ES cell cultures which subsequently produced fewer blood cells. HPC are known to actively proliferate (also observed in this study, Line 167). In complex heterogeneous culture the impact of inhibitors on EHT cannot be inferred. If expansion of blood cells inhibited, their number is reduced but the endothelial population becomes relatively enriched - but not because of suppression of EHT. The authors should track cell fate using sorted populations of interest and apply inhibitors (they did it only for some blood populations). Analysis of gene expression in total cell culture, even if snapshots are taken at a single cell level cannot tell about kinetics of EHT. Culturing sorted populations will allow them to track real and not theoretical cell-cell transitions. The analysis should be performed with clearly defined sorted hemogenic endothelial population in controlled conditions to exclude subsequent expansion of blood cells. Once protocol is established they should do single cell transcriptomics analysis to explore changes during EHT. It may be necessary to analyse EHT at a clonal level to come to a firm conclusion.

Overall, while the manuscript contains plenty of data, the approach used is somewhat superficial. The authors should have experimented with sorted populations. Although they did it for some cell fractions, they have not investigated EHT this way. The manuscript contains too many details significance and relevance of which is hard to assess. I would also expect this analysis to be performed using genetics tools since chemical inhibitors can be toxic/ affect differentiation some other ways while not necessarily killing the cells. However, they would be useful as supporting tools.

The authors should make the paper significantly more concise and focus on the data directly supporting their hypothesis.

Specific comments:
Lines 85-88: "Transcriptionally, the process is marked by the gradual downregulation of endothelial markers (e.g. CDH5, VWF) and concomitant upregulation of hemopoietic markers (e.g. RUNX1, MYB and GATA2) (Fig. 1d, e, Supplementary Fig. 1c)".
This conclusion is not supported by the data: e.g. Runx1, Gata2 and cMyb go down and not up (Fig S1). There is a number of inaccurate statements (and overstatements) of this kind scattered throughout the manuscript, which makes me unimpressed.
Line 89: "…the production of hemopoietic progenitors in vitro culminates around 3-5 days of EHT culture (Supplementary Fig. 1c)".
This figure does not show culmination of CFU but only images of methylcellulose colonies. How do they distinguish the expression of specific genes during EHT and in CFU which according to their suggestion coexist by d3? This QRT-PCR analysis does not shed light on the process, in fact it indicates that interpretation of experiments with unsorted cells is problematic.  
Line 96-112: here they describe various cell clusters identified by single cell analysis - is this really new and important? All markers that were used are well known. They need to be more specific about novelty.
Lines 113-122: "Pseudotime differentiation trajectories suggested that the hemopoietic and mesenchymal compartments both derived from a common endothelial population. Along this differentiation trajectory, endothelial cells gradually downregulated endothelial specific genes and concomitantly upregulated either mesenchymal or hemopoietic markers (Supplementary Fig. 3)".

Monocle plot does not show direction of differentiation and can be interpreted differently. It adds little to our knowledge. Additionally, violin plots in Suppl Fig 3 show specific markers in endothelial, mesenchymal and hemopoietic populations but not their "gradual" downregulation or upregulation as stated by the authors.

Lines 144- 161: hemopoietic cell fractions are sorted and tested in methylcellulose culture: different types of colonies are determined. It is unclear what this analysis gives to the main message of the paper? Many previous papers performed similar analyses with ES cells.
Line 153-161: "CDH5- HPCs could only be detected by flow cytometry, indicating that they were floating in the culture medium and were most likely derived from CDH5+ HPCs".  
It is unclear what they intended to say: "could be detected by flow" but not by microscopy? It would not be a big surprise since these two methods may have different sensitivity, depending on the antibody, fluorochromes, sensitivity of detectors. There seem to be some CDH5- cells in the CD43+ cluster shown in Fig. 3, which can belong to the CDH5- HPC.
Line 161: "Taken together, we identified a new sorting strategy to enrich for HPCs generated in vitro and confirmed their multilineage potential using differentiation assays". They have to be more specific about novelty of their sorting strategy since they used well known markers.
Lines 195-206: "Following Nocodazole treatment, cells were washed and their hemopoietic potential was assessed by CFU assays. Cell cycle inhibition effectively blocked the generation of functional hemopoietic cells (Fig. 4d)".
Here they used unsorted ES cells which contained HPC. Treatment of HPC with nocodazol for 48 hours must block their proliferation which after plating in methylcellulose will give fewer colonies.
The authors also performed "release" experiments: they incubated ES cultures with Nocodazol for 48 hours and then washed it out. They found that hemopoietic cells did not appear in this case either and concluded that proliferation of endothelial cells was necessary for EHT. However, they found that endothelial cells resumed proliferation. Why these endothelial cells, which resumed proliferation failed to generate blood cells? An explanations provided by the authors is that lack of proliferation changed their normal differentiation pathway. However, blocking polymerisation of microtubules can affect many other processes in the cell. Also, can the authors exclude a non-specific toxic effect of Nocodazol?
I also wonder about experimental setup: washing away nocodazol would probably deplete cultures from majority of Runx1+ blood cells and give same result. Did they do this washing with control cultures? If not, control cultures would contain more Runx1+ cells. If they performed washing in controls, how do they explain that CFU number here (Fig. 5b) and in previous experiments without washing (Fig. 4d) is similar?  
Lines 215-217: "The rare RUNX1+ cells detectable after treatment always displayed an endothelial morphology, as assessed by immunofluorescence and light microscopy, and were unable to acquire colony forming potential".
It is hard to see any difference in morphology of Runx1 cells in Fig. 5.
Lines 224- 328: Here the authors performed extensive analysis using specific chemical inhibitors. The majority of data represents the cell cycle status of different ES cell populations after treatment and observations on inhibition of hemopoietic cells production. These sections contain loads of small details and suggestions, which make this analysis descriptive. Making conclusions is hard since experiments were done with unsorted cells. Here they discuss that the chemicals can affect differentiation of blood cells - not just EHT. These sections are very difficult to follow.
Line 389: "However, the mesenchymal population does not appear to be affected by perturbations in the cell cycle. This would suggest that the production of mesenchymal cells is not dependent on cell cycle progression and on the activation of specific cell cycle regulators, and that this mechanism might be specific for endothelial cells undergoing EHT".
I suppose the authors imply that the endothelial to mesenchymal transition which they proposed does not require cell proliferation. However, this has a simple alternative explanation: mesenchymal cells are not generated from endothelial cells. The authors should validate their hypothesis functionally using sorted endothelial cells in culture to see if they actually produce mesenchymal cells. Reference to previous publications that suggest this would be insufficient.
 
It is also worth noting that although the relationship between proliferation and differentiation is usually considered to be inverse (e.g. Ruijtenberg and Sander van den Heuvel, Cell Cycle, 2016) some data suggest that differentiation and cell proliferation occur simultaneously but can be regulated independently: cells can start differentiating long before they stop dividing to allow cell expansion (e.g. review by Brown et al., Exp Cell Res, 2003). The authors can find these two reviews useful for planning their further analysis.

**Reviewer 2**

**Are you able to assess all statistics in the manuscript, including the appropriateness of statistical tests used?** Yes, and I have assessed the statistics in my report.

**Comments to author:**

Haemogenic endothelial cells (HEC), as defined by expression, are a population of quiescent/slow proliferating cells. This is supported in previous studies, where cells in the base of the intra-aortic haematopoietic clusters (IAHCs) are more quiescent than the more mature progenitor cells in the apex of the clusters, which are entering cell cycle in a more active manner.
The authors show that the acquisition of an active cell cycle profile allows, and results in, the specification of multipotent haematopoietic progenitor cells (HPCs), which differentiate to erythroid progenitors in their culture conditions. This is consistent with recent studies in which a cell cycle signature was not detected prior to the HSC state. The authors show that HECs subjected to a block of cell cycle, not only are unable to undergo EHT, but downregulate RUNX1 and lose their haemogenic potential and that, even upon re-establishing cell cycle activity, these cells remain with a non-haemogenic endothelial identity. This finding would suggest a narrow time window for EHT specification.
The authors show that, during EHT, quiescent HECs re-enter into cell cycle by downregulating inhibitors of the Rb and CIP/KIP families and by expressing key haematopoietic markers, marking the onset of haematopoietic specification. Given that blockage of cell cycle prevents this transition, they suggest that cell cycle regulators could be necessary for the timely expression of key haematopoietic regulators.
The authors also show that inhibition of CDK4-6 (leading to G1 arrest) as well as CDK1 (G2/M arrest) affects the transcriptional signature of the cells generated in culture and suggest a role for CDK1 (hence G2-M progression) the maintenance and survival of formed haematopoietic progenitors and for CDK4-6 (hence G1 progression) in the establishment of their differentiation potential.
Although there are not a lot of mechanistic insights and the study is mostly descriptive, I think that the paper is well written, reports an important finding and most of the data are convincing and should be published. I do, however, have some suggestions which may improve the paper. This regards mostly the last part of the manuscript that describes the inhibitor experiments.

Major comments:

1.     The authors state that their work will help to facilitate the production of human MPCs. However, for their work to be relevant to this issue, it would be important to compare the scRNA expression profiles of their in vitro EC, HEC, mesenchymal and HPC populations to those found in the human embryo. This would help understand whether their culture conditions mimic the EHT in the AGM that gives rise to HSCs or the extraembryonic generation of progenitors such as EMPs (which is likely given the CD43 and not CD45 expression and the propensity to give rise to erythroid progenitors). At least one scRNA data-set has now come out (PMID  31501518) and the data are available on GEO.
2.     According to the authors, all of the inhibitor experiments (Figs 4c, 6a,b ) were done three times, and it is stated that the figures show representatives of such experiments. Since all these experiments are quantifiable, it is important to show the variation between them in form of a bar diagram (or dots) with statistics.
3.     The single cell transcriptomics experiments that are meant to show that inhibition has also an impact on cell fate decision are definitely the weakest parts of the paper. To start with, the cut off for defining differentially expressed genes upon CDK inhibition (suppl figure 6), is only Log2 0.5 (1.41-fold). I have my doubts that these values are really significant as a cut-off should be at least two-fold, if not higher. In the absence of other data that would confirm a direct influence of CDKi on gene expression control I would therefore seriously tone down the discussion of this idea.
4.     The study also suffers from the fact that inhibitor treatment can hit cells at different phases of the cell cycle which seriously confuses the interpretation of the scRNA-Seq results. However, I appreciate that experimentally this is not a simple question to address - again, tone down the discussion and concentrate on the major findings described above such as highlighting in more detail what is remarkable about the transcriptional stage of the window in HPC development where cell cycle is necessary

Minor comments:

1.     Figure 4: To avoid having to jump around between figures, the nature of the cluster should be highlighted in Figure 4a.
2.     In general, the labelling could do with bigger letters to support the elderly population.

**Authors Response**

**Point-by-point responses to the reviewers’ comments:**

**Reviewer #1: In this study Dr Cvejic and colleagues investigated the role of cell proliferation in the endothelial-to-hemopoietic transition (EHT) in human ES cells. They found that inhibiting proliferation supresses the emergence of blood cells and concluded that it is essential for EHT. The paper contains a lot of data but is difficult to follow due to deviations and often inaccurate statements. My main concerns are:**

**(1)	The authors carried out a single cell transcriptomics analysis to infer differentiation trajectory of ES cells using Monocle software. The trajectory has 3 branches of mesodermal, endothelial and hemopoietic cells. They claim that this shows that endothelial cells generate both mesodermal and hemopoietic cells whereas in fact Monocle does not determine directions. This plot is completely compatible with the more standard view of mesodermal cells transitioning into endothelial and subsequently blood cells. The authors should validate cell fates in these 3 branches by culturing sorted populations in appropriate conditions (not just hemopoietic methylcellulose assay). I am not sure that Monocle graph and PCA analysis add any useful information to our understanding of EHT compared to what was known before. Overall this part contains a lot of details which are disentangled from the main analysis of the role of cell cycle in EHT. For example, it is not clear how pictures of cell clusters under the microscope or speculations about localisation of CDH5+ and CD5- cells in culture help understand the role of proliferation in EHT.**

The Reviewer was correct to note that Monocle did not predict the direction of the differentiation trajectory but rather implied the continuity of the differentiation process across endothelial, haematopoietic and mesenchymal cells. To address the question of the directionality and to further characterise generated cell types we performed experiments as outlined below. Of note, we did not state that endothelial cells give rise to mesodermal cells but to mesenchymal cells, through endothelial-to-mesenchymal transition (EndoMT). This process has been studied in a diversity of systems and is well described. Importantly, we agree with the Reviewer regarding the importance of this question. Indeed, an alternative possibility is that mesenchymal cells originate from a different mesodermal progenitor and are therefore already present in culture together with endothelial cells prior to EHT. Even so however, this would not affect the main conclusion of our study regarding the role of cell cycle during EHT.

To elucidate whether endothelial cells indeed give rise to both mesenchymal and haematopoietic cells we focused on characterising the initial population of CD34+/CD43- cells that we seeded at EHT D0 and we monitored the expression of CDH5, CD43 and CD44 over time. The rational here is that if the cells sorted at EHT D0 and defined as CD34+/CD43- consists of endothelial progenitors, then all other cell types that appear in culture at the later times must have originated from these endothelial progenitors.

Therefore, we performed the following experiments:

1. We have performed single-cell RNA sequencing of CD34+/CD43- cells sorted at EHT D0 (**Figure A below**). This new data demonstrated that most of the sorted CD34+/CD43- cells were endothelial cells, as shown by the positive expression of multiple endothelial marker genes (*e.g.* *CDH5*, *PECAM1*, *KDR*, *THY1*). Importantly, this data showed the absence of cells expressing haematopoietic genes at this stage, demonstrating that haematopoietic cells are subsequently generated through EHT. Interestingly, a small population of cells co-expressed endothelial markers *(e.g.* *KDR*, *THY1*) and a few mesenchymal markers (*e.g.* *COL1A1*, *CDH2*, *CDH11*, *KRT8*) at low level. This suggests that a fraction of cells displayed a mixed endothelial/mesenchymal identity at EHT D0, and it likely represented an early stage of endothelial-to-mesenchymal transition (EndoMT). The same population of cells also expressed genes indicative of an active cell cycle state (*i.e. MKI67*, *CDK1*, *HMMR)*.

2. We sorted CD34+/CD43- cells at EHT D0 and tested them in a CFU assay. Cells collected at EHT D0 were not able to generate blood colonies. This result further confirmed that at EHT D0 haematopoietic cells are not present in culture and are therefore generated afterwards.
2. We have performed time course experiments to monitor the expression of CDH5, CD43 and CD44 markers over time. This approach allowed us to further demonstrate that mature mesenchymal cells (CDH5-/CD44high/CD43-), haematopoietic progenitors HPCs (CD44+/CD43+) and erythroid cells (CD44-/CD43+), were not present at EHT D0 and are instead subsequently generated over time from the cultured endothelial cells (**Figure B below**) (as proposed in the manuscript, P1: mesenchymal cells, P4: endothelial cells, P2 and P5: erythroid cells, P3 and P6: HPCs).

4. To further confirm the identity of cells present in our *in vitro* system, at EHT D3 we sorted and re-plated endothelial and mesenchymal cells using aforementioned CD markers. After 7 days in culture, the proposed mesenchymal cells (sorted as CDH5-/CD44high/CD43- at EHT D3) displayed morphology and markers consistent with a mesenchymal identity. Specifically, these cells were CD34low/-CD90low/+CD31low/- as assessed by flow cytometry, and CDH5-/Vimentin+ as assessed by immunofluorescence. Importantly, we did not observe endothelial cells in the culture (*i.e.* all cells were CDH5- as assessed by immunofluorescence) suggesting that mesenchymal cells were not capable to differentiate to endothelial cells.

Endothelial cells (sorted as CDH5+/CD44+/CD43- at EHT D3) after 10 days in culture displayed morphology of endothelial cells. More than half of these cells was CD31high/CD90high, which is consistent with an endothelial identity. These endothelial cells were able to differentiate to new mesenchymal (CDH5-CD44high) and haematopoietic (CD43+) cells (**Figure C below**).

Overall, this additional data validated the proposed cell identities and confirmed their differentiation trajectories, demonstrating that the endothelial cells generated in this system are able to undergo EndoMT and EHT to generate mesenchymal and haematopoietic populations, respectively.

**(2)	To determine effects of cell proliferation on EHT they treated entire cultures (which contain several types of cells including hemopoietic progenitors, HPC) with various cell cycle chemical inhibitors. They observed fewer blood cells but more endothelial cells and concluded that proliferation is important for EHT. However, this might be due to inhibition of HPC expansion which already present in ES cell cultures which subsequently produced fewer blood cells. HPC are known to actively proliferate (also observed in this study, Line 167). In complex heterogeneous culture the impact of inhibitors on EHT cannot be inferred. If expansion of blood cells inhibited, their number is reduced but the endothelial population becomes relatively enriched - but not because of suppression of EHT.**

We agree that this is a fair and important point. For this reason, we performed experiments in which we focused on endothelial and HPC populations separately.

Specifically, we performed Nocodazole release experiments (Figure 5 in the manuscript), in which we focused on the effect of cell cycle block on haemogenic endothelial cells (RUNX1+ endothelial cells). In this figure, we demonstrate that regardless of its role on HPC proliferation, blocking cell cycle progression for a limited time window, followed by Nocodazole release, allows endothelial cells to reprise proliferation, showing that the cell cycle block is reversible. However, RUNX1 expression in such endothelial cells is gradually lost as a consequence. Additionally, the generation of new HPCs is not detected, also confirmed by the lack of colony forming potential for the whole culture. This figure demonstrate that endothelial cells are able to re-start proliferation after cell cycle block is removed, while their ability to undergo EHT is affected, as they downregulate RUNX1 and do not produce new haematopoietic progenitors

Furthermore, we performed CDKi experiments (Figure 6 in the manuscript) in which we treated whole cultures with inhibitors of specific cell cycle regulators, followed by HPCs isolation and culture using the same number of cells for the different conditions. This revealed that beyond their effect on HPC generation during EHT, the inhibition of CDK4/6 and CDK1 is able to impair HPC differentiation potential.

Taken together, we believe these figures address the Reviewer point, as they separately focus on endothelial and HPC populations respectively, demonstrating the role of cell cycle in endothelial cells undergoing EHT, and on HPC ability to further differentiate.

**The authors should track cell fate using sorted populations of interest and apply inhibitors (they did it only for some blood populations). Analysis of gene expression in total cell culture, even
if snapshots are taken at a single cell level cannot tell about kinetics of EHT. Culturing sorted populations will allow them to track real and not theoretical cell-cell transitions. The analysis should be performed with clearly defined sorted hemogenic endothelial population in controlled conditions to exclude subsequent expansion of blood cells. Once protocol is established they should do single cell transcriptomics analysis to explore changes during EHT. It may be necessary to analyse EHT at a clonal level to come to a firm conclusion.**

**Overall, while the manuscript contains plenty of data, the approach used is somewhat superficial. The authors should have experimented with sorted populations. Although they did it for some cell fractions, they have not investigated EHT this way.**

We believe our additional data have now addressed this point.

Briefly:

1. We have confirmed the identity of the populations generated during differentiation and validated their differentiation trajectory.
2. We have confirmed that the population sorted at EHT D0 consists of endothelial cells, and that this subsequently generates mesenchymal and haematopoietic populations.
3. Most importantly, we have shown that once sorted, endothelial cells are able to produce new mesenchymal and haematopoietic cells.

Overall, this demonstrates that endothelial cells produced in this system are a transitory population that quickly commit to mesenchymal and haematopoietic cells. Thus, performing experiments on endothelial cells after a second sorting would not solve the problem of culture heterogeneity, as these cells once re-plated would quickly produce new mesenchymal and haematopoietic cells.

However, as advised by Reviewer 1, we recognise that it is important to confirm transitions between cell states. This has now been confirmed by our new experiments, as discussed above.

**The manuscript contains too many details significance and relevance of which is hard to assess. I would also expect this analysis to be performed using genetics tools since chemical inhibitors can be toxic/ affect differentiation some other ways while not necessarily killing the cells. However, they would be useful as supporting tools.**

While the manuscript was under review we performed additional experiments taking advantage of genetic tools to further shed light on the role of CDK1 during EHT. Specifically, we generated an iPSC line that allowed us to achieve the inducible expression of a shRNA for the knockdown of CDK1. This system allowed us to demonstrate that inducing the knockdown of CDK1 has a similar effect as its chemical inhibition, depleting the colony forming potential in a CFU assay of the EHT culture (**Figure D below**).

**The authors should make the paper significantly more concise and focus on the data directly supporting their hypothesis.**

**Specific comments:**

**Lines 85-88: "Transcriptionally, the process is marked by the gradual downregulation of endothelial markers (e.g. CDH5, VWF) and concomitant upregulation of hemopoietic markers (e.g. RUNX1, MYB and GATA2) (Fig. 1d, e, Supplementary Fig. 1c)".
This conclusion is not supported by the data: e.g. Runx1, Gata2 and cMyb go down and not up (Fig S1). There is a number of inaccurate statements (and overstatements) of this kind scattered throughout the manuscript, which makes me unimpressed.**

**Line 89: "…the production of hemopoietic progenitors in vitro culminates around 3-5 days of EHT culture (Supplementary Fig. 1c)".**

**This figure does not show culmination of CFU but only images of methylcellulose colonies. How do they distinguish the expression of specific genes during EHT and in CFU which according to their suggestion coexist by d3? This QRT-PCR analysis does not shed light on the process, in fact it indicates that interpretation of experiments with unsorted cells is problematic.**

We apologise for the oversights and we have now corrected this part to more clearly reflect our data. Additionally, we have carried out minor corrections throughout the text to make the manuscript clearer and more accurate.

**Line 96-112: here they describe various cell clusters identified by single cell analysis - is this really new and important? All markers that were used are well known. They need to be more specific about novelty.**

While we agree with the Reviewer that all markers used in this study are well known, their combination is not and this combination of CD markers allowed us, for the first time, to isolate distinct populations of cells present in this *in vitro* system. Indeed, scRNA-seq data were used to devise a sorting strategy to enrich for different populations in EHT obtained *in vitro* from hPSCs. The description of the various clusters was necessary for an in-depth characterisation of the system, which was fundamental for the interpretation of subsequent data on the role of cell cycle during EHT.

**Lines 113-122: "Pseudotime differentiation trajectories suggested that the hemopoietic and mesenchymal compartments both derived from a common endothelial population. Along this differentiation trajectory, endothelial cells gradually downregulated endothelial specific genes and concomitantly upregulated either mesenchymal or hemopoietic markers (Supplementary Fig. 3)".**

**Monocle plot does not show direction of differentiation and can be interpreted differently. It adds little to our knowledge. Additionally, violin plots in Suppl Fig 3 show specific markers in endothelial, mesenchymal and hemopoietic populations but not their "gradual" downregulation or upregulation as stated by the authors.**

As described above, our new additional data allowed us to confirm the direction of the differentiation trajectory, with endothelial cells differentiating to mesenchymal and haematopoietic cells. This is in line with *in vivo* studies showing that endothelial cells generate haematopoietic cells during development, and that mesenchymal cells can be produced from endothelial cells in development and disease.

We would like to emphasise that Supplementary Figure 3a portrays only endothelial cells. All the cells composing the trajectory in this figure are endothelial cells from the initial endothelial cell cluster. Therefore, violin plots do not show expression of different genes in different populations, but instead the expression of different genes in different subsets of the endothelial cells. Therefore, we believe that our statement that “endothelial cells along the trajectory gradually downregulate endothelial specific genes and concomitantly upregulate either mesenchymal or haemopoietic markers” is correct.

**Lines 144- 161: hemopoietic cell fractions are sorted and tested in methylcellulose culture: different types of colonies are determined. It is unclear what this analysis gives to the main message of the paper? Many previous papers performed similar analyses with ES cells.**

We believe that these functional assays were important because they confirmed the identity of the HPC population. To our knowledge, this is the first time that cells generated during EHT by differentiation of hPSCs are dissected to this level, and importantly the data produced in this study allowed us to devise a strategy to enrich for different populations produced during EHT. We recognise that Figure 3 referred by Reviewer 1 does not directly address the role of cell cycle during EHT, however it represents an important characterisation of the system and a validation of the identities which were computationally assigned based on the transcriptional profiles. This is fundamental for the interpretation of the subsequent findings.

**Line 153-161: "CDH5- HPCs could only be detected by flow cytometry, indicating that they were floating in the culture medium and were most likely derived from CDH5+ HPCs".**

**It is unclear what they intended to say: "could be detected by flow" but not by microscopy? It would not be a big surprise since these two methods may have different sensitivity, depending on the antibody, fluorochromes, sensitivity of detectors. There seem to be some CDH5- cells in the CD43+ cluster shown in Fig. 3, which can belong to the CDH5- HPC.**

We are aware of the difference in sensitivity and resolution of the two techniques, and we apologise if the text was not clear enough in explaining this point. Briefly, in our experiments, we never observe CDH5- cells in the haematopoietic clusters attached to the plate, as they always appear to be CDH5+. Instead, CDH5- HPCs capable of multilineage differentiation can be detected by flow cytometry, a technique that takes into account not only the fraction of cells attached to the plate (like immunofluorescence microscopy does) but also the floating fraction, which was collected together with the attached fraction. Therefore, our data suggests that CDH5- HPCs are present in culture in the floating fraction, together with the more proliferative population of erythroid progenitors. This is consistent with *in vivo* data showing that during development the first haematopoietic cells derived through EHT express CDH5, and only later on lose this marker, leave the intra-aortic haematopoietic clusters and migrate into circulation.

**Line 161: "Taken together, we identified a new sorting strategy to enrich for HPCs generated in vitro and confirmed their multilineage potential using differentiation assays". They have to be more specific about novelty of their sorting strategy since they used well known markers.**

We have now rephrased this.

**Lines 195-206: "Following Nocodazole treatment, cells were washed and their hemopoietic potential was assessed by CFU assays. Cell cycle inhibition effectively blocked the generation of functional hemopoietic cells (Fig. 4d)".
Here they used unsorted ES cells which contained HPC. Treatment of HPC with nocodazol for 48 hours must block their proliferation which after plating in methylcellulose will give fewer colonies.**

**The authors also performed "release" experiments: they incubated ES cultures with Nocodazol for 48 hours and then washed it out. They found that hemopoietic cells did not appear in this case either and concluded that proliferation of endothelial cells was necessary for EHT. However, they found that endothelial cells resumed proliferation. Why these endothelial cells, which resumed proliferation failed to generate blood cells? An explanations provided by the authors is that lack of proliferation changed their normal differentiation pathway. However, blocking polymerisation of microtubules can affect many other processes in the cell. Also, can the authors exclude a non-specific toxic effect of Nocodazol?**

We agree with the Reviewer that non-specific toxicity of Nocodazole should be considered. For this reason, we have now included experiments in which we treated cells with Nocodazole and performed Annexin V/Propidium Iodide staining, in order to monitor if treatment increases apoptosis and cell death. Our results show that cells treated with Nocodazole only display a minor increase in Annexin V/Propidium Iodide staining, with the majority of cells being negative for both. This confirms that the observed effect is not related to increased apoptosis and allows us to exclude a non-specific toxic effect (**Figure E below**).

**I also wonder about experimental setup: washing away nocodazol would probably deplete cultures from majority of Runx1+ blood cells and give same result. Did they do this washing with control cultures? If not, control cultures would contain more Runx1+ cells. If they performed washing in controls, how do they explain that CFU number here (Fig. 5b) and in previous experiments without washing (Fig. 4d) is similar?**

In this experiment, control cells were washed the same way as cells treated with Nocodazole.

As reviewer mentioned, washes are likely to remove blood cells, especially those that have already detached and are floating in culture. However, the experiment that Reviewer is referring to was designed to monitor the effect of cell cycle on haemogenic endothelial cells (defined as RUNX1+ endothelial cells) rather than haematopoietic cells. Gentle washes do not affect attachment of endothelial cells, and they remain attached to the dish. Our results show that after washes, RUNX1+ endothelial cells treated with Nocodazole downregulate the expression of RUNX1 but retain endothelial morphology and expression of CDH5. Thus, this experiment demonstrates that blocking cell cycle affects the transition of RUNX1+ endothelial cells towards haematopoietic progenitors.

As for the number of colonies in CFU assay, in Figure 4d in the manuscript cells were collected at EHT D5. Considering that haematopoietic clusters start to appear at EHT D3, the system had 2 days to produce HPCs. In Figure 5b instead, cells were washed at EHT D5, which as noted by the Reviewer is likely to remove any HPC generated. However, after release, cells were allowed to recover for 48h before being collected for CFU assay at EHT D7, as described in figure legend. Again, the system had 2 days to produce new HPCs, which might account for the similar number of colonies observed in CFU assay in Figure 4c and in Figure 5b.

**Lines 215-217: "The rare RUNX1+ cells detectable after treatment always displayed an endothelial morphology, as assessed by immunofluorescence and light microscopy, and were unable to acquire colony forming potential".
It is hard to see any difference in morphology of Runx1 cells in Fig. 5.**

We have now edited the figure and outlined the haematopoietic clusters.

Briefly, in CTRL at EHT D5 and EHT D6, we see many RUNX1+ cells that are mainly round shaped and grouped in clusters. These clusters can also be recognised in the CDH5 stained panel (green), in which they display a typical round morphology, as opposed to the surrounding elongated endothelial cells. Finally, these clusters can also be recognised in the DAPI (grey) panel, in which they are characterised by the typical dense nuclear morphology and brighter appearance compared to endothelial cells.

In Nocodazole treated cultures, all these features are not present: the few RUNX1+ cells are more scattered and not grouped in clusters; they mark elongated endothelial cells rather than round cells; these elongated cells are CDH5 positive (green panel) and thus clearly endothelial; finally, their nuclei do not appear to be denser or brighter than the surrounding cells, as was the case in haematopoietic clusters found in control.

**Lines 224- 328: Here the authors performed extensive analysis using specific chemical inhibitors. The majority of data represents the cell cycle status of different ES cell populations after treatment and observations on inhibition of hemopoietic cells production. These sections contain loads of small details and suggestions, which make this analysis descriptive. Making conclusions is hard since experiments were done with unsorted cells. Here they discuss that the chemicals can affect differentiation of blood cells - not just EHT. These sections are very difficult to follow.**

We have now edited the text and simplified the description.

**Line 389: "However, the mesenchymal population does not appear to be affected by perturbations in the cell cycle. This would suggest that the production of mesenchymal cells is not dependent on cell cycle progression and on the activation of specific cell cycle regulators, and that this mechanism might be specific for endothelial cells undergoing EHT".**

**I suppose the authors imply that the endothelial to mesenchymal transition which they proposed does not require cell proliferation. However, this has a simple alternative explanation: mesenchymal cells are not generated from endothelial cells. The authors should validate their hypothesis functionally using sorted endothelial cells in culture to see if they actually produce mesenchymal cells. Reference to previous publications that suggest this would be insufficient.**

As described above, we have now performed experiments to functionally demonstrate that mesenchymal cells derive from endothelial cells.

**It is also worth noting that although the relationship between proliferation and differentiation is usually considered to be inverse (e.g. Ruijtenberg and Sander van den Heuvel, Cell Cycle, 2016) some data suggest that differentiation and cell proliferation occur simultaneously but can be regulated independently: cells can start differentiating long before they stop dividing to allow cell expansion (e.g. review by Brown et al., Exp Cell Res, 2003). The authors can find these two reviews useful for planning their further analysis.**

We thank the reviewer for the insight. We and others have shown that differentiation can occur concomitantly with cell cycle progression of hPSCs and that cell cycle regulators play an active role in cell fate decision (Pauklin et al., Cell 2013, Genes and Dev 2016, Yangiou et al., JBC 2019). The current manuscripts extend those results to EHT and suggests that cell cycle progression is again necessary for differentiation. Nonetheless, we agree that differentiation and cell cycle progression appear to have different relationships depending on the context *e.g.* cell type and system used. For this reason, we believe that our work would be of general interest for the field as we focused on examining the relationship between differentiation and cell cycle progression during EHT and developmental haematopoiesis.

**Reviewer #2: Haemogenic endothelial cells (HEC), as defined by expression, are a population of quiescent/slow proliferating cells. This is supported in previous studies, where cells in the base of the intra-aortic haematopoietic clusters (IAHCs) are more quiescent than the more mature progenitor cells in the apex of the clusters, which are entering cell cycle in a more active manner.
The authors show that the acquisition of an active cell cycle profile allows, and results in, the specification of multipotent haematopoietic progenitor cells (HPCs), which differentiate to erythroid progenitors in their culture conditions. This is consistent with recent studies in which a cell cycle signature was not detected prior to the HSC state. The authors show that HECs subjected to a block of cell cycle, not only are unable to undergo EHT, but downregulate RUNX1 and lose their haemogenic potential and that, even upon re-establishing cell cycle activity, these cells remain with a non-haemogenic endothelial identity. This finding would suggest a narrow time window for EHT specification.
The authors show that, during EHT, quiescent HECs re-enter into cell cycle by downregulating inhibitors of the Rb and CIP/KIP families and by expressing key haematopoietic markers, marking the onset of haematopoietic specification. Given that blockage of cell cycle prevents this transition, they suggest that cell cycle regulators could be necessary for the timely expression of key haematopoietic regulators.
The authors also show that inhibition of CDK4-6 (leading to G1 arrest) as well as CDK1 (G2/M arrest) affects the transcriptional signature of the cells generated in culture and suggest a role for CDK1 (hence G2-M progression) the maintenance and survival of formed haematopoietic progenitors and for CDK4-6 (hence G1 progression) in the establishment of their differentiation potential.
Although there are not a lot of mechanistic insights and the study is mostly descriptive, I think that the paper is well written, reports an important finding and most of the data are convincing and should be published. I do, however, have some suggestions which may improve the paper. This regards mostly the last part of the manuscript that describes the inhibitor experiments.

Major comments:**

**1.	The authors state that their work will help to facilitate the production of human MPCs. However, for their work to be relevant to this issue, it would be important to compare the scRNA expression profiles of their in vitro EC, HEC, mesenchymal and HPC populations to those found in the human embryo. This would help understand whether their culture conditions mimic the EHT in the AGM that gives rise to HSCs or the extraembryonic generation of progenitors such as EMPs (which is likely given the CD43 and not CD45 expression and the propensity to give rise to erythroid progenitors). At least one scRNA data-set has now come out (PMID  31501518) and the data are available on GEO.**

We are grateful for the Reviewer’s kind comments and suggestions. As suggested, we have now performed comparative analysis between our data and the publicly available *in vivo* data obtained from AGM (Zeng *et al.*; doi.org/10.1038/s41422-019-0228-6) (**Figure F below**).

Briefly, the dataset derived from *in vivo* AGM was analysed and annotated according to the annotations found in Table S3 and in Figures 1-4 from Zeng *et al.* This allowed us to generate a UMAP space with the Authors’ original cell annotations. Having confirmed that cells of similar cell type are clustered together, forming well defined clusters, we then mapped this dataset onto our data. This allowed us to obtain predictions of the cell identities in our data using the annotations from the *in vivo* generated dataset (**Figure F, a**). This confirmed that endothelial, mesenchymal and haematopoietic cells identified in our dataset displayed similarity with corresponding populations described *in vivo*.

Finally, we used marker genes for distinct cell populations highlighted in Figure 1-4 from Zeng *et al.* and assessed their expression in the clusters we previously identified (**Figure F, b**)

Here are few important findings:

1. Similarly to AGM-derived haemogenic endothelial cells, endothelial cells generated in our system appeared to express both haemogenic genes and arterial genes, confirming their developmental arterial origin.
2. Our endothelial cells show higher similarity with late haemogenic endothelial cells (late HECs) compared to early haemogenic endothelial cells (early HECs) from AGM. According to Zeng *et al.*, true HSPCs are generated only from the late HECs.
3. Zeng *et al.* describes 3 types of HSPCs at different stages of maturation. HSPC1 are proposed to be early haematopoietic cells, HSPC2 are characterised by genes consistent with an active cell cycle, and HSPC3 are suggested to be more mature cells expressing stem cell markers such as *GFI1*. By comparing the expression of top marker genes expressed in these populations, we concluded that genes marking HSPC1 are mainly expressed in our endothelial cells, while our HPC cluster displays similarities with the HSPC3 population with the addition of cell cycle related genes as in HSPC2.

Overall, this comparison shows that endothelial cells generated in our study are similar to haemogenic endothelial cells found in human AGM, with higher similarity to late haemogenic endothelium; furthermore, it suggests that our population of *in vitro* derived HPCs displays similarities with end stage HSPCs obtained *in vivo*, while still retaining features of immature cells such as an active cell cycle. Therefore, the analysis suggest that our *in vitro* system is able to recapitulate populations found in the human AGM and provide a confirmation that the mechanisms described in this study are relevant for human development.

As suggested by the Reviewer we have also checked for the expression of CD45 (*PTPRC*) in our HPCs, confirming that the gene is indeed expressed.

**2.	According to the authors, all of the inhibitor experiments (Figs 4c, 6a,b ) were done three times, and it is stated that the figures show representatives of such experiments. Since all these experiments are quantifiable, it is important to show the variation between them in form of a bar diagram (or dots) with statistics.**

We have now produced the plot as advised and included it in the relevant figure.

**3.	The single cell transcriptomics experiments that are meant to show that inhibition has also an impact on cell fate decision are definitely the weakest parts of the paper. To start with, the cut off for defining differentially expressed genes upon CDK inhibition (suppl figure 6), is only Log2 0.5 (1.41-fold). I have my doubts that these values are really significant as a cut-off should be at least two-fold, if not higher. In the absence of other data that would confirm a direct influence of CDKi on gene expression control I would therefore seriously tone down the discussion of this idea.**

**4.	The study also suffers from the fact that inhibitor treatment can hit cells at different phases of the cell cycle which seriously confuses the interpretation of the scRNA-Seq results. However, I appreciate that experimentally this is not a simple question to address - again, tone down the discussion and concentrate on the major findings described above such as highlighting in more detail what is remarkable about the transcriptional stage of the window in HPC development where cell cycle is necessary**

As advised by the Reviewer, we have edited these paragraphs.

**Minor comments:

1.	Figure 4: To avoid having to jump around between figures, the nature of the cluster should be highlighted in Figure 4a.**

**2.	In general, the labelling could do with bigger letters to support the elderly population.**

We have edited the figures following Reviewer 2 suggestions.

**Second round of review**

**Reviewer 2**

The authors have addressed all questions and have significantly improved the paper
